# Neuroprotective Potential of Indole-Based Compounds: A Biochemical Study on Antioxidant Properties and Amyloid Disaggregation in Neuroblastoma Cells

**DOI:** 10.3390/antiox13121585

**Published:** 2024-12-23

**Authors:** Tania Ciaglia, Maria Rosaria Miranda, Simone Di Micco, Mariapia Vietri, Gerardina Smaldone, Simona Musella, Veronica Di Sarno, Giulia Auriemma, Carla Sardo, Ornella Moltedo, Giacomo Pepe, Giuseppe Bifulco, Carmine Ostacolo, Pietro Campiglia, Michele Manfra, Vincenzo Vestuto, Alessia Bertamino

**Affiliations:** 1 Department of Pharmacy, University of Salerno, Via G. Paolo II, 84084 Fisciano, Italy; tciaglia@unisa.it (T.C.); mmiranda@unisa.it (M.R.M.); mvietri@unisa.it (M.V.); gsmaldone@unisa.it (G.S.); smusella@unisa.it (S.M.); vdisarno@unisa.it (V.D.S.); gauriemma@unisa.it (G.A.); csardo@unisa.it (C.S.); moltedo@unisa.it (O.M.); gipepe@unisa.it (G.P.); bifulco@unisa.it (G.B.); costacolo@unisa.it (C.O.); pcampiglia@unisa.it (P.C.); abertamino@unisa.it (A.B.); 2 NBFC—National Biodiversity Future Center, 90133 Palermo, Italy; 3 European Biomedical Research Institute of Salerno (EBRIS), Via Salvatore de Renzi 50, 84125 Salerno, Italy; s.dimicco@ebris.eu; 4 Department of Health Science, University of Basilicata, Viale dell’Ateneo Lucano 10, 85100 Potenza, Italy

**Keywords:** neuroprotection, amyloid, disaggregation, antioxidants, indole nucleus, in-cell studies

## Abstract

Based on the established neuroprotective properties of indole-based compounds and their significant potential as multi-targeted therapeutic agents, a series of synthetic indole–phenolic compounds was evaluated as multifunctional neuroprotectors. Each compound demonstrated metal-chelating properties, particularly in sequestering copper ions, with quantitative analysis revealing approximately 40% chelating activity across all the compounds. In cellular models, these hybrid compounds exhibited strong antioxidant and cytoprotective effects, countering reactive oxygen species (ROS) generated by the Aβ(25–35) peptide and its oxidative byproduct, hydrogen peroxide, as demonstrated by quantitative analysis showing on average a 25% increase in cell viability and a reduction in ROS levels to basal states. Further analysis using thioflavin T fluorescence assays, circular dichroism, and computational studies indicated that the synthesized derivatives effectively promoted the self-disaggregation of the Aβ(25–35) fragment. Taken together, these findings suggest a unique profile of neuroprotective actions for indole–phenolic derivatives, combining chelating, antioxidant, and anti-aggregation properties, which position them as promising compounds for the development of multifunctional agents in Alzheimer’s disease therapy. The methods used provide reliable in vitro data, although further in vivo validation and assessment of blood–brain barrier penetration are needed to confirm therapeutic efficacy and safety.

## 1. Introduction

Oxidative stress (OS) is characterized by an imbalance between reactive oxygen species (ROS) or reactive nitrogen species (RNS) production and the body’s antioxidant defense mechanisms, represented by catalase and superoxide dismutase enzymes or non-enzymatic factors, such as glutathione, urate, and bilirubin [1,2,3,4]. This imbalance leads to cellular injury and subsequent dysfunctions that can result in a wide range of disorders, such as neurodegenerative ones, including Alzheimer’s disease (AD), Parkinson’s disease, Amyotrophic Lateral Sclerosis, and Huntington’s disease [5,6,7,8,9,10]. When reactive species production exceeds the capacity of these defenses, oxidative stress ensues, leading to membrane lipid peroxidation, protein misfolding, and alterations in cellular functions, which in turn can trigger neuroinflammation. Although neuroinflammation at controlled levels serves as a marker of development and plays a protective role, its excessive or chronic activation contributes to cellular damage and exacerbates neurodegenerative processes [11,12,13]. Eventually, the persistent OS and neuroinflammation can trigger multiple cell death pathways, including apoptosis, necrosis, necroptosis, pyroptosis, ferroptosis, and autophagy [14,15,16,17,18,19].

OS is thereby intricately connected in the pathophysiology of AD. ROS generated by amyloid fibrils can trigger critical inflammatory signaling pathways, resulting in the increased production of pro-inflammatory cytokines, the aggregation of amyloid peptides into fibrils, and the subsequent formation of plaques. This interaction initiates a harmful feedback loop, in which inflammation amplifies ROS production, worsening oxidative stress and sustaining the neuronal damage [20,21,22,23]. Moreover, the potential of β-amyloid (Aβ) peptides to induce OS is also associated with the complexes it forms with redox active metals [24]. The binding of iron, zinc, and copper to Aβ has been established to promote its aggregation into plaques. Among these metals, copper forms the most stable complexes with Aβ, generating superoxide and hydrogen peroxide [25,26,27,28]. The resulting oxidative stress caused by the metal–amyloid complexes has been shown to further exacerbate amyloid protein accumulation in the extracellular space of the brain, which ultimately harms neurons. This process triggers an intracellular protein misfolding, which disrupts protein degradation systems and results in endoplasmic reticulum (ER) stress. The subsequent cellular dysfunction contributes to excitotoxicity, synaptic impairment, mitochondrial dysfunction, inflammation, DNA damage, and inappropriate reactivation of the cell cycle, culminating in cell death [29,30,31,32,33].

Employing antioxidants is a widely recognized and thoroughly researched strategy for countering oxidative stress and safeguarding human health. These molecules act as protective agents, neutralizing oxidants before they can damage vital biomolecules, essentially serving as sacrificial compounds in the process [34,35,36,37,38,39].

Beyond natural antioxidants, which have attracted significant interest as co-adjuvants for the treatment of many pathologies, synthetic molecules with similar “scavenger” properties are also worthy of the same scientific interest. The main chemical requirement for this class of compounds is represented by a high electronic availability needed for the ROS and RNS detoxification. In synthesizing such a kind of molecules, the scaffold choice is critical, and it should be based primarily on chemical versatility and economic feasibility. For these reasons, we considered the indole ring, an aromatic heterocycle containing nitrogen atoms that is recognized as a privileged scaffold [40,41,42,43,44] in medicinal chemistry. Indole rings are present in a variety of naturally occurring compounds as well as in many synthetic pharmaceuticals, showing diverse pharmacological roles, such as their ability to function as anticancer, anti-inflammatory, disaggregating, and antimicrobial agents [45,46,47,48,49,50,51,52,53].

Hence, the use of an indole-privileged scaffold takes advantage of the drug design process to develop new agents targeting the interconnected pathways of misfolded proteins and neuroinflammation [54,55,56,57].

Based on the well-documented versatility of the indole nucleus and the widely described capability of phenol fragments to behave as multifunctional antioxidants [58,59,60,61], we were prompted to test a series of hybrid compounds endowed with an indole–phenol structure. Inspired by our previous results indicating the absence of a significant cytotoxic activity [62] by the indole–phenolic compounds, we decided to test them as potential AD neuroprotectors. Focused on the desired outcome, we selected different indoles with amine-chelating groups at positions 3 and 5 to evaluate their influence on the antioxidant and disaggregating activities. Their antioxidant and disaggregating activities were tested through a variety of assays, and their neuroprotective effects were analyzed in the AD model represented by Aβ(25–35) neuronal aggregates in the SH-SY5Y cell line.

The innovative aspect of this approach lies in the design of multifunctional compounds that combine metal-chelating, antioxidant, and protein-disaggregating properties, which could target multiple interrelated pathways involved in AD. The advantages of this strategy include the potential for synergistic effects, offering a more comprehensive treatment option for neurodegenerative diseases. However, challenges remain, particularly regarding the need for further in vivo validation, as well as overcoming issues related to bioavailability and efficient blood–brain barrier penetration for therapeutic effectiveness.

## 2. Materials and Methods

### 2.1. Chemistry

Unless otherwise stated, all reagents and solvents used were purchased from Sigma-Aldrich (Milan, Italy). Reactions were performed under magnetic stirring in round-bottom flasks. TLC analysis of reaction mixtures was performed on pre-coated glass silica gel plates (F254, 0.25 mm, VWR International, Radnor, PA, USA). The crude products were purified by the Isolera Spektra One automated flash chromatography system (Biotage, Uppsala, Sweden) using commercial silica gel cartridges (SNAP KP-Sil, Biotage). NMR spectra were recorded at room temperature on a Bruker Avance 400 MHz apparatus. Chemical shifts were reported in δ values (ppm) relative to internal Me_4_Si for ^1^H and ^13^C NMR. J values were reported in hertz (Hz). ^1^H NMR peaks were described using the following abbreviations: s (singlet), bs (broad singlet), d (doublet), t (triplet), and m (multiplet). HR-MS spectra were obtained using an LTQ-Orbitrap-XL-ETD mass spectrometer (Thermo Scientific, Bremen, Germany), equipped with electrospray ionization. The purity of the final compounds was determined through ultra-high-performance liquid chromatography (UHPLC) on a Jasco Extrema LC 4000 system. This system included an LC-Net CG cable controller, a quaternary flow pump (PU-4285), a DG-4000–04 degasser, a UV-4075 detector, and an AS-4250 autosampler (Jasco, Tokyo, Japan). UHPLC purity assessments were carried out using an EVO C18 column (150 mm × 2.1 mm × 2.6 μm, 100 Å; Phenomenex, Bologna, Italy). The mobile phase was optimized with 0.1% HCOOH in H_2_O (A) and 0.1% HCOOH in ACN (B). Gradient elution conditions were as follows: 0–10 min, 5–95% B; 10–12 min, 95% B; 12–15 min, isocratic at 5% B. The flow rate was maintained at 0.5 mL/min, and the injection volume was 5 μL.

### 2.2. Sample Preparation for Analyses and Circular Dichroism Studies

Before performing experiments, Aβ(25–35) peptide (Sigma Aldrich, St. Louis, MO, USA) was prepared by dissolving it in PBS (pH 7.4) at a concentration of 2.5 mM. Subsequently, the molecules (30 µM) were incubated with Aβ(25–35) (40 µM) for 24 h.

Circular dichroism (CD) spectra were recorded using a JASCO J-810 spectropolarimeter (Jasco, Tokyo, Japan) equipped with a 1 mm pathlength quartz cell, operated at 25 °C. The CD data were collected as an average of four scans over a wavelength range of 260–190 nm, with a bandwidth of 1 nm and a scanning speed of 10 nm/min. Solvent spectra were subtracted from each measurement to obtain the final spectra. CD curve analysis was conducted using the CONTIN algorithm available on the DICHROWEB online platform [63,64].

### 2.3. Thioflavin T Assay

Aβ(25–35) aggregation was performed with thioflavin T (ThT, Sigma Aldrich, St. Louis, MO, USA) staining. Aβ(25–35) (40 µM) was aggregated for 24 h in PBS (pH 7.4) at 37 °C under slow agitation. Then, compounds (30 µM) were added into 96-well plates together with Aβ(25–35) for 24 h. Aβ(25–35) (40 µM) alone was used as a positive control. Following the washing step, a staining solution comprising 20 µM ThT in PBS was applied and incubated for 30 min at 37 °C in the absence of light. Fluorescence signals (excitation at 450 nm and emission at 482 nm) were measured using a PerkinElmer EnSpire multimode plate reader and recorded as ThT fluorescence intensity [65].

### 2.4. Metal Chelating Experiment

Metal binding studies were conducted following the protocol outlined in reference [66]. The UV absorption spectra of the compounds (30 μM) were measured either alone or after incubation with CuSO_4_, FeSO_4_, or ZnCl_2_ (40 μM) for 30 min in a solution containing 20% (*v*/*v*) ethanol and buffer (20 mM HEPES, 150 mM NaCl, pH 7.4). Measurements were performed using a Multiskan Go microplate reader (Thermo Scientific, Waltham, MA, USA) over a wavelength range of 280 to 400 nm. The total reaction volume was 1 mL.

### 2.5. Determination of Copper-Chelating Activity

The copper chelation activity was assessed following the method of Kubglomsong et al. [67]. In brief, a 4 mM solution of the chromogenic reagent pyrocatechol violet was prepared in 50 mM sodium acetate buffer (pH 6.0). In a 96-well plate, 10 µL of the compounds (30 µM), 70 µL of sodium acetate buffer, 10 µL of CuSO_4_ (1 mM), and 4 µL of pyrocatechol violet were added. The mixture was thoroughly mixed, and the absorbance of the pyrocatechol–Cu^2+^ complex was measured at 632 nm using a Multiskan Go spectrophotometer (Thermo Scientific, Waltham, MA, USA). Ethylenediaminetetraacetic acid (EDTA, final concentration 1 mM) was used as a positive control. The results are expressed as the chelating activity of compounds.

### 2.6. Cell Culture

The human neuroblastoma cell line SH-SY5Y was sourced from the American Type Culture Collection (ATCC, Rockville, MD, USA). Cells were cultured in Dulbecco’s Modified Eagle’s Medium (DMEM) containing 4500 mg/mL glucose, supplemented with 10% (v/v) fetal bovine serum, 2 mM L-glutamine, 100 U/mL penicillin, and 0.1 mg/mL streptomycin. Cultures were maintained in Corning culture dishes (Corning, NY, USA) under a 95% humidified atmosphere with 5% CO_2_ at 37 °C and were subcultured every 2 days. All experiments were conducted using cells between passages 17 and 20. For each experiment, the cells were transferred into fresh medium and treated with compounds at varying concentrations and incubation times, as detailed in the following sections. Each treatment and analysis were conducted across three independent experiments.

### 2.7. MTT Assay

The mitochondrial metabolic activity was established by using MTT (3-(4,5-Dimethylthiazol-2-yl)-2,5-diphenyltetrazolium bromide) [68]. Briefly, SH-SY5Y (5 × 10^4^ cells/well) were plated in 96-well plates, and then compounds (30 µM) were added for 24 h alone or together with H_2_O_2_ and Aβ(25–35). H_2_O_2_ (500 µM) and Aβ(25–35) (40 µM) alone were used as positive controls.

Afterward, the MTT reagent (0.5 mg/mL) was added and incubated for 1 h. Then, 100 μL per well of 0.1 M isopropanol/HCl solution was added to solubilize formazan. The absorbance was measured at 570 nm using a microplate reader (Multiskan Go, Thermo Scientific, Waltham, MA, USA). Cell viability was expressed as a percentage of untreated cells cultured in medium with 0.1% DMSO, which was set to 100%, whereas 10% DMSO was used to set 0% viability. The EC_50_ values were calculated using GraphPad Prism 8.0 software by nonlinear regression of the dose–response inhibition.

### 2.8. Measurement of LDH

To verify the release of LDH into the cell culture medium after plasma membrane disruption, the LDH-Glo^TM^ cytotoxicity assay (Promega, Fitchburg, WI, USA) was performed. SH-SY5Y cells were seeded (5 × 10^4^ cells/well) in a 96-well plate, allowing them to adhere for 24 h. Afterward, the cells were incubated for 24 h with compounds (30 µM) and Aβ(25–35) (40 µM). According to the LDH-Glo^TM^ kit protocol, the LDH detection reagent (containing lactate, NAD^+^, reductase, reductase substrate, and rLuciferase Ultra-Glo^TM^) was added to the cell culture medium samples. The luminescent signal generated was read in end point mode using a PerkinElmer AlphaScreen multimode plate reader (PerkinElmer, Waltham, MA, USA) [66].

### 2.9. ROS Determination

The levels of reactive oxygen species (ROS) were evaluated using 6-carboxy-2′,7′-dichlorodihydrofluorescein diacetate (DCFH-DA, 10 μM, Sigma Aldrich, St. Louis, MO, USA). SH-SY5Y cells were seeded at a density of 5 × 10^4^ cells per well in a black 96-well ViewPlate (PerkinElmer, Waltham, MA, USA) and allowed to adhere for 24 h. Subsequently, cells were treated with compounds (30 µM) and H_2_O_2_ for 24 h, with H_2_O_2_ (800 µM for 1 h) serving as a positive control. After washing, the cells were incubated with DCFH-DA in serum-free medium without phenol red for 30 min at 37 °C in the dark. Fluorescence signals (excitation at 485 nm and emission at 535 nm) were measured using a PerkinElmer EnSpire multimode plate reader and expressed as the DCFH fluorescence intensity [69].

### 2.10. Thioflavin T In-Cell Imaging

Aβ(25–35) aggregation was also analyzed by using in-cell thioflavin T (ThT, Sigma Aldrich, St. Louis, MO, USA) staining by making minor changes to the previous protocol [66]. SH-SY5Y cells (5 × 10^4^ cells/well) were grown in 96-well plates and allowed to adhere for 24 h. Later, the medium was replaced, and the cells were treated with compounds (30 µM) and also Aβ(25–35) (40 µM) as the positive control for 24 h. After treatments, the culture medium was replaced, and the cells were washed and suspended in ThT (final concentration, 20 µM) at 37 °C for 20 min in the dark. The stained cells were washed, and representative images were acquired using a ZOE Fluorescent Cell Imaging System (Magnification, 20×). Quantitative analyses were performed reading the fluorescence signals (excitation/emission 450 nm/482 nm) using a PerkinElmer EnSpire multimode plate reader (PerkinElmer, Waltham, MA, USA).

### 2.11. Statistical Analysis

The results are presented as the mean ± standard deviation (SD) from three separate experiments. Statistical evaluation was conducted using analysis of variance (ANOVA), followed by Bonferroni’s post hoc test for multiple comparisons, utilizing GraphPad Prism 8.0 software (San Diego, CA, USA). Significance was assumed at *p* < 0.05.

### 2.12. Molecular Docking

The structures of **12**–**14** and **20**–**22** were sketched by Build Panel of Maestro (version 11, New York, NY, USA) and their geometries were refined by applying the OPLS3 force field [70,71], the Polak–Ribier conjugate gradient algorithm (PRCG, 9 × 10^7^ steps, maximum derivative less than 0.001 kcal/mol), and GB/SA (Generalized Born/surface area) solvent treatment [72] of H_2_O. Next, the small molecules were processed by means of LigPrep generating the protonation states at pH = 7.0 ± 1.0 [73]. The model of Aβ(25–35) was built by Build Panel of Maestro (version 11, New York, NY, USA), building the first undecameric strand and processed by Protein Preparation Wizard. This strand was then replicated to obtain a total of ten copies. These monomers were flanked by taking into account the averaged distance (1.7 Å) between interstrand NH-O, observable from deposited experimental structures [74,75]. The so-built model was refined by two rounds of hybrid Monte Carlo of the Prime [76,77] module. The first round was executed using the default parameters, except for the number of steps, which was set to 100, and the number of output structures, which was set to 50. Cartesian constraints were applied to the peptide backbone with 10.0 kcal/(molÅ^2^) and a distance constraint of 0 Å. The obtained best structure was used as the input conformation for a second run by means of the same parameters except for the distance of 0.5 Å in the cartesian constraints. Glide software [78,79,80,81] was employed for docking calculations. The blind docking was carried out by centering the grid between the two A30 of chains E and F, along with inner and outer boxes of 10 Å and 36 Å, respectively. Firstly, one docking calculation with Standard Precision (SP) was employed to yield one pose per ligand applying default parameters plus enhanced sampling for conformer generation and expanded sampling for initial pose selection. The obtained docked poses were employed as input conformations for three runs of Extra Precision (XP) mode predictions: adding to the default parameters—the enhanced sampling mode, maintaining 10,000 poses/ligand for the initial docking step, 1000 poses/ligand for energy minimization, and 1000 maximum output structures/ligand. Throughout the calculations, only the trans conformation of the amide bond was allowed. Moreover, the intramolecular H-bond reward, aromatic bonds, and Epik state penalty were considered as energy contributions. The same set of docking calculations was executed with a grid sized for the inner/outer box as 10/31 Å and centered on the following x, y, and z cartesian coordinates: −8.71, −12.2, and 8.47. The analysis of the docking outcome and figure preparation were conducted in Maestro (version 11, New York, NY, USA).

### 2.13. Molecular Dynamics

The Aβ(25–35) model, both in its the free and **20**-bound states, was used for the molecular dynamics simulation. These input models were created using the System Builder in Desmond with the following setup: an orthorhombic box with a 15 Å buffer distance, the OPLS3 force field, the TIP3P [82,83] solvation model, Cl^−^ and Na^+^ ions for electroneutrality, and a NaCl solution (0.15 M). Initially, the molecular systems were optimized using the LBFGS method with default parameters, followed by a relaxation as described: (i) NVT simulation with restrained solute heavy atoms (1 ns, 10 K, small time steps); (ii) NVT simulation with restrained solute heavy atoms (120 ps, 10 K; Berendsen thermostat); (iii) NPT simulation (120 ps, 10 K) restraining the solute heavy atoms with Berendsen thermostat and Berendsen barostat (1 atm), fast temperature relaxation constant, slow pressure relaxation constant, and velocity resampling of 1 ps; (iv) restrained solute heavy atom NPT ensemble simulation (120 ps) applying a Berendsen barostat (1 atm) and Berendsen thermostat (310 K), fast temperature relaxation constant, slow pressure relaxation constant, and velocity resampling of 1 ps; and (v) a 240 ps NPT simulation was conducted using the Berendsen thermostat (310 K) and Berendsen barostat (1 atm), with normal pressure and rapid temperature relaxation constants. Unrestrained molecular dynamics were performed for 100 ns at 310 K with an NPT (1.01 bar) ensemble. The simulation employed a recording time of 1.2 ps and an integration time step of 2.0 fs. As a reference, we also repeated the simulation applying backbone positional restraints (force constants = 0.001 kcal/mol/Å^2^). The pressure, volume, temperature, and total and potential energies were monitored for each equilibration step through the Simulation Quality Analysis tool of Desmond.

### 2.14. Binding Site Investigation

SiteMap [84,85] calculations were performed using default parameters, with both less and more restrictive definition of hydrophobicity, and fine grid. For AutoSite [86] analysis, the grid boxes were centered as reported above and sized as 47 × 54 × 35 Å along the x, y, and z axes, with the grid points spaced at 1.0 Å. For the ligand atom types, the C, HD, and OA maps were calculated.

## 3. Results

### 3.1. Chemical Synthesis

**Figure 1:**
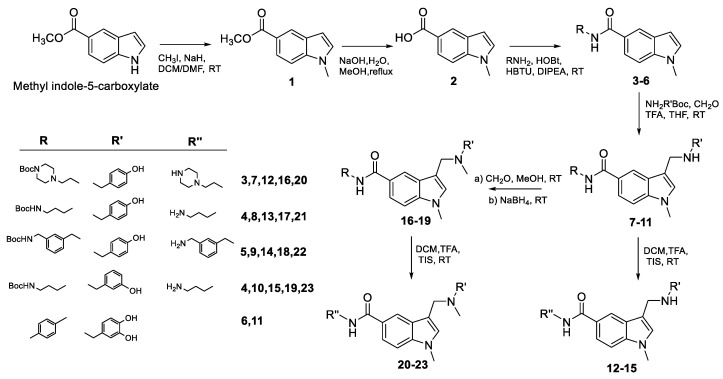
Scheme of the synthesized molecules.

The synthesis of the final derivatives **11**, **12**–**15**, and **20**–**23** was performed as reported in Figure 1 following synthetic routes previously reported in our work [62]. Methyl indole-5-carboxylate was reacted with methyl iodide in a basic medium, obtaining intermediate **1** in 84% yield. Subsequentially hydrolysis under basic conditions generated **2** (94% yield), which was treated with different protected and unprotected amines via a coupling reaction, leading to intermediates **3**–**6** (62–70% yield). The obtained compounds were further derivatized at position 3 by a Mannich reaction, employing various substituted hydroxybenzylamines, trifluoroacetic acid, and formaldehyde as the methylene group donor, generating **7**–**11** (44–55% yield). Intermediates **7**–**10** were deprotected at position 3 using a mixture of DCM/TFA and triisopropylsilane as scavengers, leading to the final derivatives **12**–**15** in almost quantitative yields. Compounds **7**–**10** were further substituted at position 3, introducing a methyl group by a reductive amination reaction in the presence of formaldehyde and NaBH_4_ as a reducing agent, yielding the tertiary amines **16**–**19** (40–52% yield). N-Boc removal, under the conditions described above, furnished the final derivatives **20**–**23** almost quantitatively.

### 3.2. Biochemistry Investigation

#### 3.2.1. Chelating Properties

**Figure 2:**
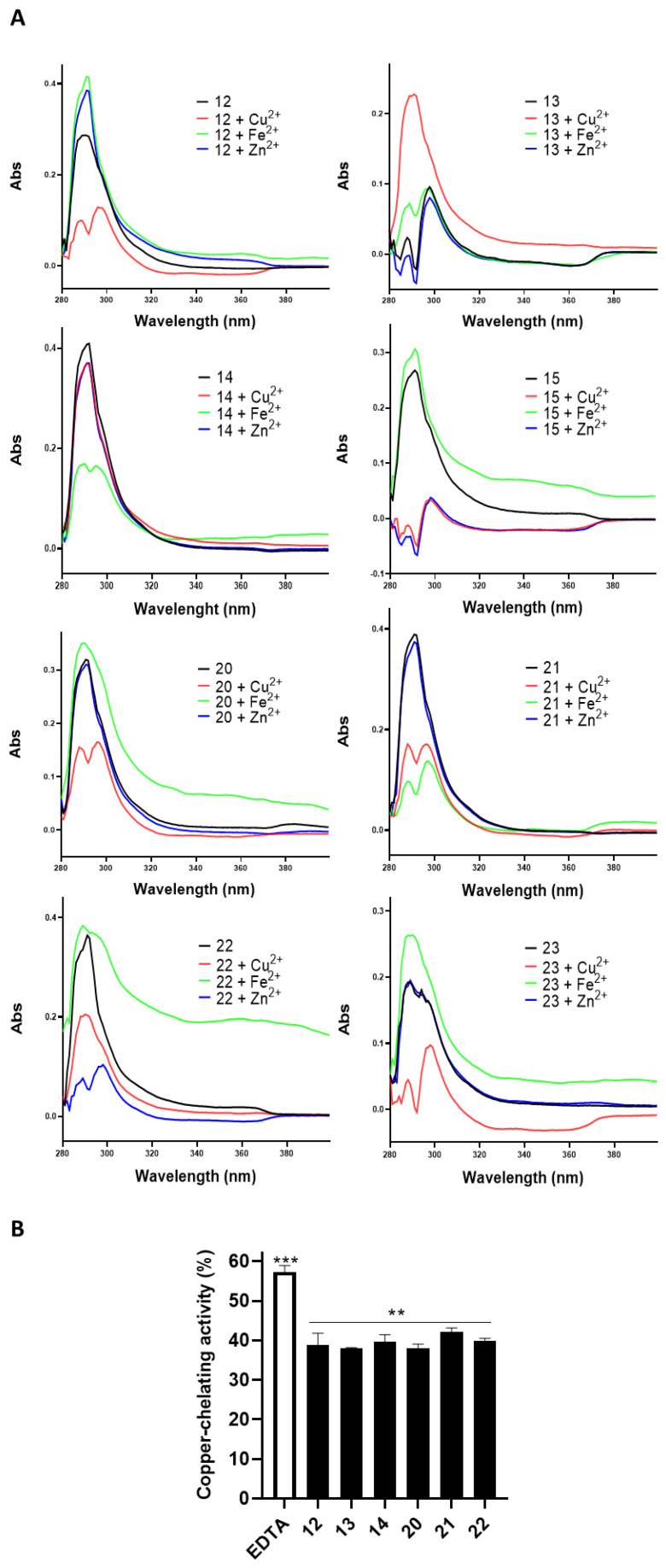
(**A**) UV spectra (in the range of 280 to 400 nm) of compounds (30 µM) alone and in the presence of 40 µM FeSO_4_, FeCl_3_, and CuSO_4_. (**B**) Copper-chelating quantitative analyses of compounds (30 µM). EDTA (1 mM) was used as the positive control. Results are shown as mean ± standard deviation (SD) from three independent experiments. **, *** denote *p* < 0.01 and *p* < 0.001 vs. Ctrl.

The ability of compounds to chelate biometals such as Cu(II), Fe(II), and Zn(II) was studied by means of UV-VIS spectroscopy. Upon the addition of CuSO_4_ to a solution with the compounds, the absorbance decreased dramatically, indicating the formation of a strong molecule–Cu(II) complex. The absorption maximum at 230 nm showed a slight shift when FeSO_4_ or ZnCl_2_ was added, suggesting that the compounds also bind Fe(II) and Zn(II) (Figure 2A). EDTA was used as a positive control (Figure S25). Finally, these results demonstrate that the compounds could exert antioxidant activity through the chelation of iron, zinc, and copper metal ions.

Given the possible chelating properties of compounds, particularly vs. copper, the pyrocatechol violet assay was selected for the next experiment to enable the accurate quantification of copper ions. The results demonstrated a significant capacity of the compounds to effectively bind with copper ions (**12**: 38.93 ± 2.90%; **13**: 38.09 ± 0.14%; **14**: 39.68 ± 1.67%; **20**: 38.09 ± 0.98%; **21**: 42.12 ± 1.06%; **22**: 39.96 ± 0.57%; *p* < 0.05 vs. Ctrl; EDTA: 57.32 ± 1.63%; *p* < 0.01 vs. Ctrl).

#### 3.2.2. In-Cell Neuroprotection

The antioxidant activity of the compounds was then evaluated in cells. First, the molecules were analyzed to assess their effect on SH-SY5Y cell viability.

**Figure 3:**
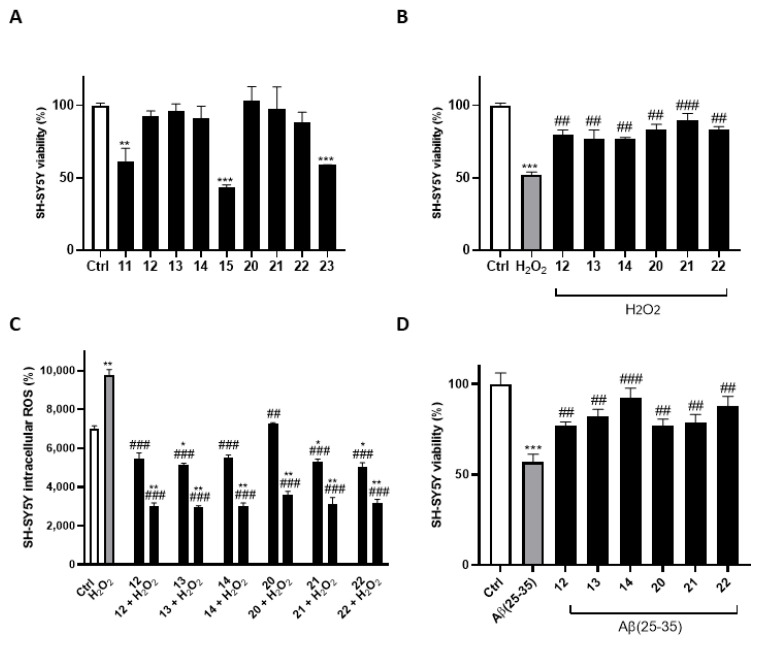
Neuroprotective activity of compounds. (**A**) SH-SY5Y cells were exposed to compounds at a concentration of 30 µM. Neuroprotective effects of compounds against (**B**) H_2_O_2_-induced (500 μM) cytotoxicity, (**C**) H_2_O_2_-induced (500 μM) ROS production, and (**D**) Aβ(25–35)-induced (40 μM) cytotoxicity. The 2′,7′-dichlorofluorescin diacetate (DCFH-DA) assay was conducted to reveal ROS production. The changes in viability were determined by calculating the percentage of viable cells in treated cultures relative to untreated controls. The results are presented as the mean ± standard deviation (SD) from three independent experiments. *, **, and *** denote, respectively, *p* < 0.05, *p* < 0.01, and *p* < 0.001 vs. Ctrl; ^##^, and ^###^ denote, respectively, *p* < 0.01, and *p* < 0.001 vs. H_2_O_2_ or Aβ(25–35).

Our experimental results showed that most of our compounds (final concentration, 30 μM) did not lead to a decrease in cell viability in the tested cell line after 24 h of treatment except for compounds **11** (*p* < 0.01 vs. Ctrl), **15** (*p* < 0.001 vs. Ctrl), and **23** (*p* < 0.001 vs. Ctrl) (Figure 3A). For this reason, such compounds were excluded from subsequent analyses.

H_2_O_2_ is a common ROS that can be used to mimic oxidative stress conditions in in vitro studies. By introducing H_2_O_2_, it is possible to simulate the oxidative environment associated with amyloid aggregation and neurodegenerative diseases such as AD [25,87,88,89]. To assess the protective effects of the compounds against oxidative stress, we first conducted an MTT assay using H_2_O_2_-stimulated SH-SY5Y cells. After 24 h, H_2_O_2_ induced a significant reduction in metabolic activity (52.28 ± 1.77% cell viability, *p* < 0.001 vs. Ctrl). The compounds significantly reduced cell mortality in the presence of H_2_O_2_ compared to cells treated with H_2_O_2_ alone, thus preserving cell viability and demonstrating their neuroprotective properties (**12**: 79.98 ± 3.15%; **13**: 76.93 ± 6.11%; **14**: 76.18 ± 0.74%; **20**: 83.69 ± 3.22%; **22**: 83.59 ± 1.83%; *p* < 0.01 vs. H_2_O_2_. **21**: 89.41 ± 5.03%; *p* < 0.001 vs. H_2_O_2_) (Figure 3B).

Subsequently, we corroborated the antioxidant activity results by performing a ROS assay in the H_2_O_2_-induced oxidative stress model. The results showed that H_2_O_2_ significantly increased ROS production in SH-SY5Y cells (*p* < 0.01 vs. Ctrl). When the compounds were added to the cells, the release of ROS was significantly inhibited (**12**–**14**, **20**–**22**: *p* < 0.001 vs. H_2_O_2_), thus confirming the involvement of the antioxidant properties of the molecules in the neutralization of ROS after 24 h of treatment (Figure 3C).

We then proceeded to the next step, in which we tested the compounds on an Aβ cell model to further evaluate their potential protective effects. As shown in Figure 3D, cell viability in the Aβ group decreased to 56.78 ± 4.35% compared to the Ctrl group (*p* < 0.001), suggesting that Aβ(25–35) is cytotoxic to SH-SY5Y cells. After treatment with the compounds, the cell viability increased significantly compared to the Aβ(25–35) group (**12**: 76.85 ± 2.17%; **13**: 81.79 ± 4.11%; **20**: 76.15 ± 3.43%; **21**: 78.54 ± 4.58%; **22**: 87.86 ± 5.34%; *p* < 0.01 vs. Aβ; **14**: 92.50 ± 5.13%; *p* < 0.001 vs. Aβ).

Furthermore, to corroborate the neuroprotection against Aβ(25–35)-induced damage in SH-SY5Y cells, an LDH assay was performed alongside MTT. The results confirmed that the compounds not only increased mitochondrial activity but also significantly reduced LDH release, demonstrating their ability to mitigate neuroinflammation (Figure S24).

These results indicate that compounds have the potential to provide neuroprotective effects against Aβ-induced cytotoxicity in vitro.

#### 3.2.3. Investigation on Disaggregating Properties of Compounds

**Figure 4:**
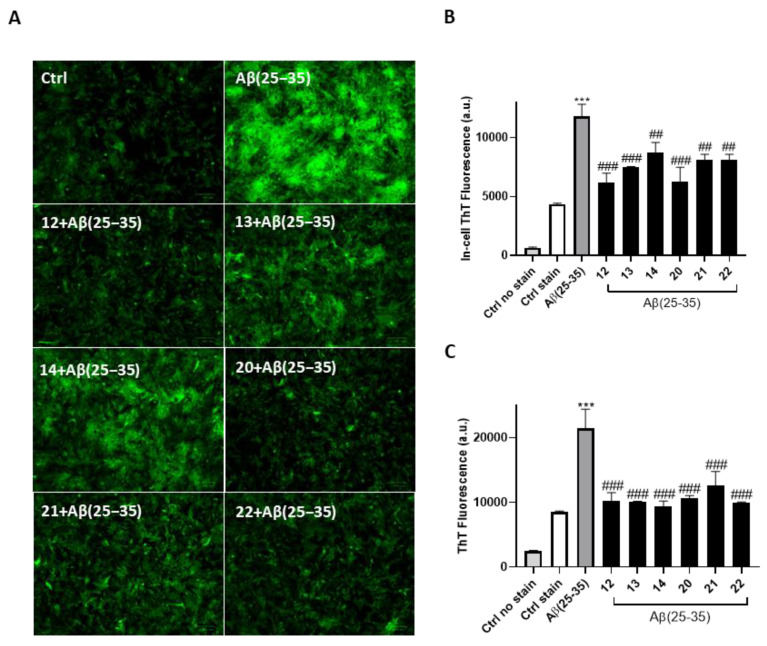
Disaggregating properties of compounds. In-cell ThT assay was performed for both fluorescence microscopy (**A**) and spectrophotometry (**B**). Scale bar: 100 μm. (*N* ≥ 10). Cells were observed at 20× magnification. (**C**) ThT shows a direct disaggregating effect of compounds against Aβ. Data are shown as mean ± SD of three different experiments performed in triplicate. *** denotes *p* < 0.001 vs. Ctrl; ^##^, and ^###^ denote, respectively, *p* < 0.01, and *p* < 0.001 vs. H_2_O_2_ or Aβ(25–35).

The ability of the synthesized compounds **12**–**14** and **20**–**22** to disaggregate Aβ(25–35) fibrils was evaluated using a thioflavin T (ThT) assay. An in vitro model was commonly employed to explore the cellular and molecular mechanisms underlying neurological disorders, as well as for preliminary drug screening in AD. The compounds were tested at concentrations of 30 μM alongside 40 μM Aβ(25–35) before being aggregated for 24 h. The thioflavin T fluorescence intensity readings after 24 h of incubation were compared with those of the control sample containing only Aβ(25–35) (*p* < 0.001 vs. Ctrl). The quantitative analysis showed that the treatments with 30 μM concentrations of compounds led to a marked reduction in fluorescence intensity compared to Aβ(25–35) (**12**, **13**, **20**: *p* < 0.001 vs. Aβ; **14**, **21**, **22**: *p* < 0.001 vs. Aβ) (Figure 4B).

In addition, fluorescence images of ThT-treated cells were acquired. ThT selectively accumulated in amyloid deposits, thereupon exhibiting a dramatic increase in fluorescent brightness in contrast with a less florescent brightness in the cells treated with the tested compounds, in accordance with the quantitative analyses (Figure 4A).

**Figure 5:**
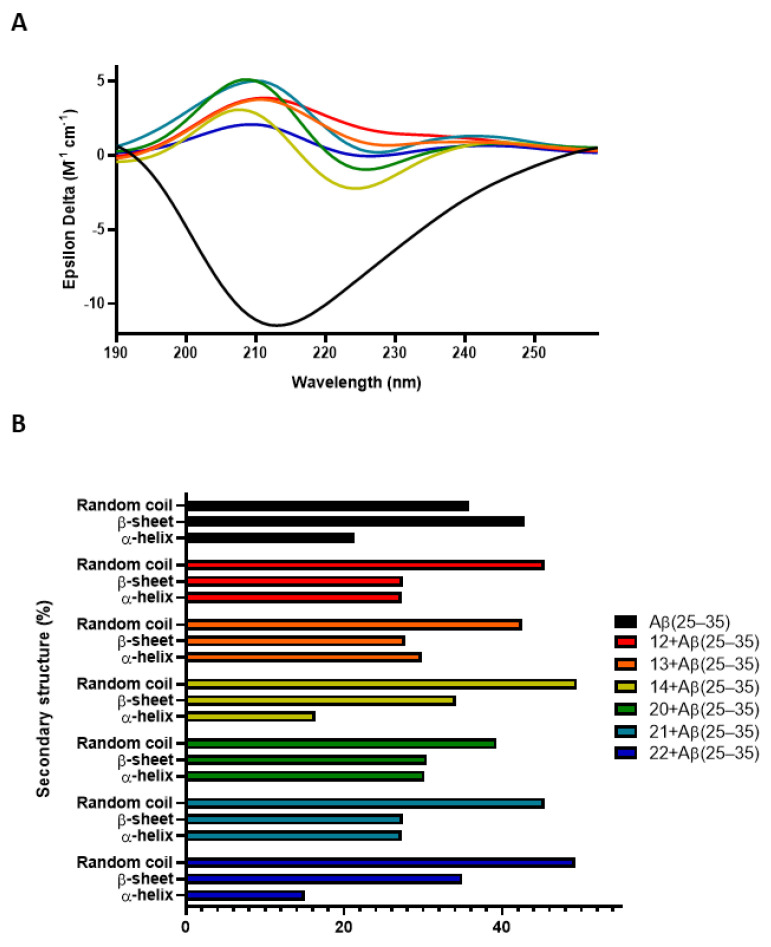
(**A**,**B**) CD curves and secondary structure analysis of the Aβ(25−35) peptide were performed using the CONTIN algorithm after 24 h of aggregation. Aβ(25–35) 40 μM was used as a positive control. A different colour has been selected for Aβ(25–35) and each compound in presence Aβ(25–35), as indicated in the legend.

After demonstrating these interesting in-cell disaggregating properties, we next investigated the mechanisms by which the molecules may act on amyloid fibrils to promote disaggregation. A ThT assay was conducted to evaluate the direct disaggregating effect of compounds incubated with Aβ(25–35). Our findings demonstrated a direct disaggregating effect on Aβ (**12**–**14**, **20**–**22**: *p* < 0.001 vs. Aβ) (Figure 4C), while no effects were observed on amyloid peptide aggregation inhibition (Figure S2), confirming a selective mechanism. At this stage, circular dichroism studies were conducted to confirm and further explore the disaggregation mechanism. Figure 5 shows the CD spectra of Aβ(25–35) after 24 h of incubation of both the peptide alone and with compounds. The extended polyproline conformation of the Aβ peptide displays a trend toward a positive CD signal around 215 nm typically indicating a precursor of β-sheet formation in line with the literature [90,91,92]. Accordingly, CONTIN analysis (Figure 5B) indicates that Aβ(25–35) alone has 48.50% β-sheet conformation and 35% random coil conformation. In contrast, the peptide incubated with the molecules shows a significant reduction in β-sheet conformation (**12**: 27.40%; **13**: 27.70%; **14**: 34.20%; **20**: 30.50%; **21**: 27.40%; **22**: 35.00%). At the same time, an increase in the random coil conformation was also found in almost all the treatments, so demonstrating the compounds’ disaggregating activity.

### 3.3. Computational Studies

**Figure 6:**
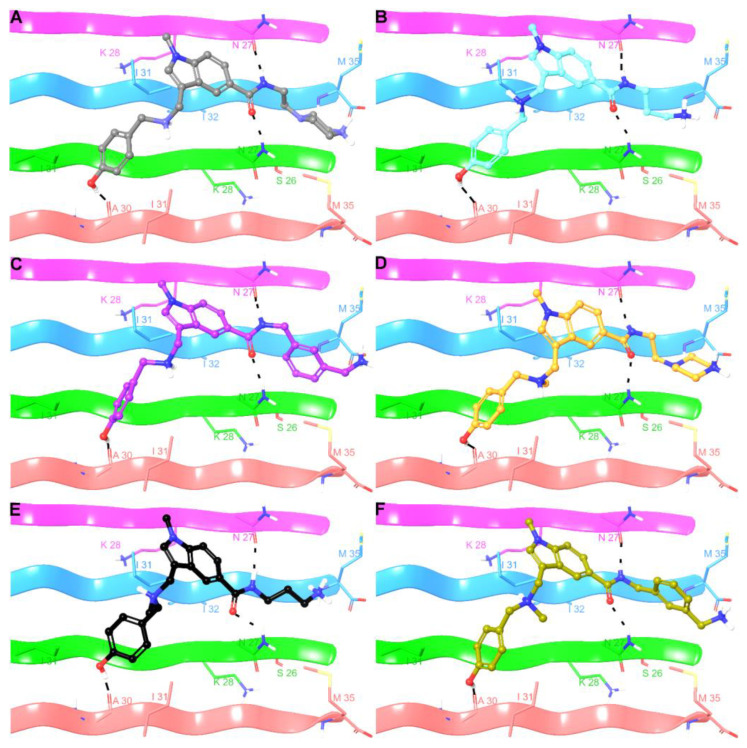
Three-dimensional model of the interactions of **12** (**A**), **13** (**B**), **14** (**C**), **20** (**D**), **21** (**E**), and **22** (**F**) with Aβ(25–35). The biological target is depicted by a ribbon colored by a chain (D, magenta; E, azure; F, green; G, faded red) and tube (colored: C, by chain; polar H, white; N, dark blue; O, red; S, yellow). The small molecules are represented by sticks (gray for **12**, cyan for **13**, violet for **14**, orange for **20**, black for **21**, khaki for **22**) and balls (colored: C, as for the sticks; polar H, white; N, dark blue; O, red). The hydrogen bonds between the ligand and Aβ(25–35) are represented by the dashed black lines.

Given that the experimental data suggested the disaggregating properties of the indole-based compounds, we investigated their interactions with Aβ(25–35) by molecular modeling calculations [93], with the goal of identifying the structural elements responsible for the binding of small molecules to the biological target and for designing more potent analogs. As the experimental structures of Aβ(25–35) in the oligomeric/protofibril/fibril [94,95] state were not reported in the protein data bank, we constructed a representative theoretical model (see Section 2 for further details). Specifically, as suggested by experimental studies [96,97,98,99], we built an antiparallel β-sheet structure. As binding site information was not available, the first step of our structural investigation was a blind docking [100,101,102] calculation with a grid encompassing the whole target. We observed that the docked poses converged towards the β-sheet face opposite to the protruding charged lysine residues, as also corroborated by the top-ranked site identified by SiteMap and AutoSite. This was probably due to the electrostatic repulsion with protonated amine groups, accounting for the physiological pH, of **12**–**14** and **20**–**22**. Moreover, we observed that the molecules interact essentially with the central region of the β-sheet spanning from chain C to H. Based on these results, we proceeded with a second round of docking calculations considering a narrowed conformational space. The docking results showed that **12**–**14** and **20**–**22** shared a similar binding pose (Figure 6). The phenol portion donates a hydrogen bond to the backbone CO of A30, and the aromatic moiety is surrounded by three side chains of I31, leading to van der Waals interactions. The amide group of the small molecules establishes two H-bonds with side chain of two N27, while the amine groups form a salt bridge with the carboxylate function of M35. The N-methyl indole moiety provides van der Waals contacts with I31 and donates an aromatic H-bond to the side chain of N27. The piperazine rings of **12** and **20** and benzylamine of **14** also contribute to the affinity against the biological target by van der Waals contacts with M35. The N-methyl groups of **20** and **22** also established van der Waals contacts with I31.

As all ligands share the same docked poses and showed comparable experimental results, we used compound **20** as a representative ligand for molecular dynamics calculations (100 ns, 310 K). The overall intermolecular interactions between **20** and Aβ(25–35), observed from molecular docking, were kept during the trajectory (>30%) [103], along with the atom-relative orientation of **20** (Figure S3) [104,105,106]. Moreover, the comparison of the trajectories of Aβ(25–35) in the free and bound state (Figure S4) would suggest that the ligand induces destabilization of the biological target especially after 50 ns, but further studies should be performed.

## 4. Discussion

The current therapeutic options for Alzheimer’s disease, primarily acetylcholinesterase inhibitors, are limited and provide only modest symptom relief [107,108]. As a result, there is significant interest among medicinal chemists in developing new Aβ disaggregating agents with broadened biological properties, such as antioxidative effects and metal-chelating capabilities in order to harness the therapeutic efficacy [28,109,110,111,112,113].

In this work, a series of 1,3,5-trisubstituted indole derivatives was synthesized by linking the heterocyclic indole moiety with different phenol and amine fragments. The synthetic pathway was mainly based on the derivatization of methyl indole-5-carboxylate via Mannich and coupling reactions, resulting in good yields and purity of the target compounds.

Establishing a safety profile is a crucial step in the early evaluation of potential drug candidates. Encouragingly, almost all of the indole hybrids exhibited minimal to no cytotoxicity when tested against the SH-SY5Y cell line, suggesting they have favorable safety and biocompatibility profiles. The SH-SY5Y human neuroblastoma cell line is widely recognized in neuroscience research for its ability to mimic several characteristics of human neurons. These include the expression of key enzymes such as dopamine-β-hydroxylase and tyrosine hydroxylase, as well as high levels of axonally localized tau protein, making it an invaluable model for studying nervous system development and function [111,114,115,116,117]. Furthermore, it can be used to study different neurogenerative disorders, such as Alzheimer’s and Parkinson’s disease [118,119,120]. In particular, in vitro models of AD used Aβ(25–35) to initiate neurotoxicity in various cultured cell types, including undifferentiated SH-SY5Y cells [121,122,123,124,125].

In the present study, we provided direct evidence that highlights how our compounds protect SH-SY5Y cells against Aβ(25–35)-induced cytotoxicity, discussing many aspects. Firstly, we demonstrated the chelating properties of the synthesized compounds against different transition metal ions, including zinc, copper, and iron. It is widely known that such polyvalent metal cations, particularly copper, are found in high concentrations in senile plaques in AD patients’ brain and have been shown to strongly modulate the self-assembly of the Aβ peptides into insoluble fibrils modulating their cytotoxicity [126,127,128,129,130,131,132]. Aβ possesses selective high- and low-affinity binding sites for Cu²⁺ and Zn²⁺, which mediate its resistance to protease degradation and reversible precipitation [133,134,135]. These metals also contribute to oxidative damage, with Cu²⁺ driving the oxygen-dependent production of H_2_O_2_, exacerbating Aβ toxicity. These metal–Aβ interactions not only promote amyloid aggregation but also enhance oxidative stress, a major contributor to neuronal injury [136,137,138]. Importantly, studies show that Cu/Zn chelators can solubilize Aβ from postmortem AD brain tissue [139], highlighting the therapeutic potential of metal chelation operating through two possible pathways: mitigating oxidative damage and reducing amyloid aggregation, making it a promising therapeutic strategy in AD.

Then, the antioxidant properties of the nontoxic derivates **12**–**14** and **20**–**22** were tested, with the results showing a significant inhibition of cell death induced by Aβ(25–35) and its bioproduct H_2_O_2_, strongly blocking ROS release, increasing the mitochondrial activity and cell viability, as well as reducing LDH release. ROS may attack cell membrane lipids, alter proteins, and damage nucleic acids. Consequently, numerous neurons in the hippocampus and cortex, being unable to counteract oxidative imbalance, are likely to undergo significant cell death, a defining feature of AD and other neurodegenerative disorders [140,141]. Studies have clearly shown that Aβ toxicity is linked to a rise in intracellular ROS levels, contributing to cellular damage and dysfunction [142,143,144]. OS can also trigger the expression and improper processing of amyloid precursor protein, which then produces amyloidogenic fragments [145]. This vicious cycle may become self-sustaining, as OS promotes amyloid production, and Aβ(25–35) in turn escalates OS, leading to neuronal imbalance and, ultimately, cell death. Studies have demonstrated that antioxidant therapies could offer significant promise in managing AD, as they may interrupt this harmful cycle and protect neurons from oxidative damage [146,147]. Moreover, the neuroprotective effects of indole-based antioxidants have been explored in various preclinical models, showing reduced amyloid burden, improved cognitive function, and preserved neuronal integrity [148,149,150].

In this context, the compounds tested in this study present a promising strategy, as they demonstrate the ability to reduce ROS production, suggesting that their protective effects may be, at least in part, due to the suppression of oxidative stress pathways. These findings align with the existing literature, which highlights the therapeutic potential of modulating oxidative stress for combating neurodegenerative diseases, particularly in AD. Further studies are warranted to explore the long-term efficacy and mechanistic pathways involved in their antioxidant action, potentially paving the way for novel treatments aimed at halting or reversing neurodegeneration. We further explored the mechanism underlying the protective effect of indole derivatives against Aβ(25–35)-induced in SH-SY5Y cells by evaluating their ability to reduce the amyloid misfolded peptide accumulation. All the compounds, especially compounds **12** and **20**, demonstrated strong potential as modulators of Aβ(25–35) misfolding, consistent with the ThT fluorescence assay results. To determine whether these modulators act as disaggregating agents or inhibit aggregation, multiple ThT assays were conducted both before and after Aβ(25–35) peptide aggregation. The results indicated that these compounds exhibit a robust ability to disaggregate amyloid peptides rather than prevent their aggregation.

To support these data, circular dichroism studies were conducted by incubating the peptide with the compounds. The results revealed a significant reduction in β-sheet conformation, accompanied by an increase in random coil conformation across nearly all treatments, indicating the compounds’ disaggregating activity.

These findings were integrated by a molecular modelling investigation to identify key structural features responsible for binding towards Aβ(25–35). Firstly, our analysis showed that the compounds **12**–**14** and **20**–**22** preferentially targeted the β-sheet face opposite to that one with exposed charged lysines, likely due to electrostatic repulsion with the protonated amine groups in the small molecules. Molecular docking demonstrated a shared binding pose and the key intermolecular interactions, identifying binding hot spots. Specifically, a network of three hydrogen bonds was identified by the phenol and amide groups with A30 and N27, respectively, along with a salt-bridge by the terminal amine with the carboxylate group of M35. Compound-specific features, such as the piperazine in **12** and **20**, or phenyl in **14** and **22** further contributed to binding by Van der Waals contacts. The observed interactions were maintained over time as shown by molecular dynamics with the **20**-Aβ(25–35) complex, and the simulations also indicate the potential structural destabilization of Aβ after 50 ns that may explain the compound’s disaggregating activity.

Reducing β-sheet conformations in amyloid is vital for preventing protein aggregation, a major contributor to toxicity. Amyloid fibrils and their oligomers harm neurons, altering the permeability of cell membranes, leading to the opening of calcium channels and disruption of calcium homeostasis. This surge in intracellular calcium levels triggers a cascade of events that can result in OS and neuronal damage [151,152,153,154,155,156]. Therefore, decreasing β-sheet levels may mitigate this toxicity and protect neuronal function, which is often impaired by amyloid accumulation.

In summary, this study highlights the potential of 1,3,5-trisubstituted indole derivatives as multifunctional neuroprotectors for Alzheimer’s disease. The compounds demonstrated strong antioxidant, metal-chelating, and Aβ(25–35) disaggregating activities, which protect SH-SY5Y cells from Aβ(25–35)-induced cytotoxicity. The results suggest that these compounds may mitigate oxidative stress, inhibit amyloid aggregation, and potentially reduce neuroinflammation, offering a promising approach for AD therapy. The advantage of this strategy lies in the multifunctional nature of the compounds, targeting multiple pathological mechanisms of AD. However, this study has some limitations, including the reliance on in vitro models, which may not fully reflect the complexity of AD in vivo. Additionally, further investigations into the pharmacokinetics and long-term efficacy of these compounds are needed to assess their potential for therapeutic use. Overall, this study provides valuable insights into the design of novel therapeutic agents but emphasizes the need for further validation and optimization.

## 5. Conclusions

AD is a multifactorial disorder characterized by interconnected underlying mechanisms, including OS, mitochondrial dysfunction, neuronal loss, inflammation, and abnormal protein aggregation. In line with this complexity, multitarget therapeutic strategies are increasingly being explored to address the various pathological pathways involved in neurodegeneration. In this work, new indole-based compounds have been synthesized and biologically tested as potential neuroprotectors, exhibiting antioxidant, chelating, and disaggregating properties. The in vitro neuroprotective effects were initially evaluated by H_2_O_2_- and Aβ(25–35)-induced oxidative stress on SH-SY5Y cells, showing antioxidant and cytoprotective effects. To further explore the disaggregating potential of these compounds, we conducted ThT fluorescence assays, both in cell cultures and on isolated compounds incubated with Aβ peptide. This comprehensive approach allowed us to assess the compounds’ efficacy in inducing Aβ disaggregation. The results were further validated through circular dichroism analysis, which confirmed the compounds’ ability to disrupt the β-sheet conformation of the amyloid peptide. Computational analyses further corroborated the disaggregating properties observed in previous assays, revealing that the indole derivatives interact with Aβ(25–35) by forming stable molecular interactions, including hydrogen bonds, salt bridges, and van der Waals contacts. Molecular dynamics simulations using compound **20** as a representative ligand indicated that these interactions were largely maintained over the 50 ns simulation period. Notably, the simulations suggested that the ligand induces destabilization of the Aβ(25–35) structure, supporting the previously obtained results regarding the compounds’ ability to promote the disaggregation of the Aβ peptide. These findings reinforce the potential of our compounds as promising templates in the development of multifunctional agents for Alzheimer’s therapy, capable of targeting OS, metal chelation, and amyloid disaggregation. However, further in vivo studies and pharmacokinetic evaluations are necessary to fully assess their therapeutic efficacy and clinical applicability.

## Data Availability

The original contributions presented in this study are included in the article/Supplementary Materials. Further inquiries can be directed to the corresponding authors.

## Supplementary Materials

The following supporting information can be downloaded at: https://www.mdpi.com/article/10.3390/antiox13121585/s1, Figure S1: Thioflavin T fluorescence microscopy raw images; Figure S2: Thioflavin T fluorescence assay; Figure S3: Contact histograms during the simulation of 20-Aβ(25-35) model; Figure S4: Backbone RMSD (Å) plot of Aβ(25-35) model; Figure S5: The Root Mean Square Fluctuation (RMSF) of side chains of Aβ(25-35) model; Figure S6: 1H NMR spectra of compound 11; Figure S7: DEPT spectra of compound 11; Figure S8: 1H NMR spectra of compound 12; Figure S9: DEPT spectra of compound 12; Figure S10: 1H NMR spectra of compound 13; Figure S11: DEPT spectra of compound 13; Figure S12: 1H NMR spectra of compound 14; Figure S13: DEPT spectra of compound 14; Figure S14: 1H NMR spectra of compound 15; Figure S15: DEPT spectra of compound 15; Figure S16: 1H NMR spectra of compound 20; Figure S17: DEPT spectra of compound 20; Figure S18: 1H NMR spectra of compound 21; Figure S19: DEPT spectra of compound 21; Figure S20: 1H NMR spectra of compound 22; Figure S21: DEPT spectra of compound 22; Figure S22: 1H NMR spectra of compound 23; Figure S23: DEPT spectra of compound 23; Figure S24: LDH assay performed on SH-SY5Y cells; Figure S25: UV spectra (in range 280 to 400 nm) of positive ctrl EDTA.
