# Supplementary material for: Neuroprotective Potential of Indole-Based Compounds: A Biochemical Study on Antioxidant Properties and Amyloid Disaggregation in Neuroblastoma Cells
Source: Antioxidants (Basel). 2024 Dec 23;13(12):1585. doi: 10.3390/antiox13121585 (PMC11673510; doi:10.3390/antiox13121585)
Supplement: Supplementary file 1 [file antioxidants-13-01585-s001.zip › antioxidants-3339509-supplementary.pdf]

Supporting Information

Neuroprotective Potential of Indole-Based Compounds: A Biochemical Study on Antioxidant Properties and Amyloid Disaggregation in Neuroblastoma Cells

Tania Ciaglia,[#,1] Maria Rosaria Miranda,[#,1,2] Simone Di Micco,[3] Mariapia Vietri,[1] Gerardina Smaldone,[1] Simona Musella,[1] Veronica Di Sarno,[1] Giulia Auriemma, [1] Carla Sardo, [1] Ornella Moltedo,[1] Giacomo Pepe,[1] Giuseppe Bifulco,[1] Carmine Ostacolo,[1] Pietro Campiglia,[1] Michele Manfra,[*,4] Vincenzo Vestuto,[*,1] and Alessia Bertamino[1]

[1] Department of Pharmacy, University of Salerno, Via G. Paolo II, Fisciano, 84084 Salerno, Italy; tciaglia@unisa.it, mmiranda@unisa.it, mvietri@unisa.it, gsmaldone@unisa.it, smusella@unisa.it, vdisarno@unisa.it, csardo@unisa.it, gauriemma@unisa.it, moltedo@unisa.it, gipepe@unisa.it, bifulco@unisa.it, costacolo@unisa.it, pcampiglia@unisa.it, vvestuto@unisa.it, abertamino@unisa.it

[2] NBFC—National Biodiversity Future Center, 90133 Palermo, Italy; mmiranda@unisa.it, gipepe@unisa.it

[3] European Biomedical Research Institute of Salerno (EBRIS), Via Salvatore de Renzi 50, 84125, Salerno, Italy; s.dimicco@ebris.eu

[4] Department of Health Science, University of Basilicata, Viale dell'Ateneo Lucano 10, 85100 Potenza, Italy; michele.manfra@unibas.it

* Correspondence: michele.manfra@unibas.it; vvestuto@unisa.it

\# These authors contributed equally

Chemical procedures and NMR characterization of compounds

General procedure A: coupling reaction (3-6)

Intermediate **2** (1.0 mmol) was dissolved in dichloromethane and added with different aromatic or aliphatic amines (1.2 mmol), HOBt (1.2 mmol), HBTU (1.2 mmol) and DIPEA (2.4 mmol) and the mixture was stirred at room temperature overnight. Then, the mixture was diluted with dichloromethane (20 mL), and the resulting solution was washed successively with 10% aqueous solution of citric acid (2 × 25 mL), 10% aqueous solution of $NaHCO_3$ (2 × 25 mL), and water (2 × 25 mL), dried over Na_2SO_4, filtered and concentrated under vacuum. Flash chromatography of the residues, using mixtures of ethyl acetate/n-hexane as mobile phase yielded the intermediates 3-6 in 65-75% of yield.

General procedure B: Mannich reaction (7-11)

Formaldehyde (2.0 eq), trifluoroacetic acid (2.0 eq) and 4-hydroxybenzylamine, 3-hydroxybenzylamine or 3,4-dihydroxybenzylamine (2.0 eq) were dissolved in tetrahydrofuran and the solution was stirred at room temperature for 30 min. Afterwards, the opportune 1,5-disubstituted indole derivative (1.0 mmol) was added and the mixture was stirred for the further 4-6 h. After the

completion of the reaction monitored by TLC, the reaction was quenched by 10% aqueous solution of sodium bicarbonate, diluted with dichloromethane, separated, dried over anhydrous Na_2SO_4 and filtered. The organic layer was evaporated in vacuo and 1,3,5-substituted indole derivatives 7-11 were obtained after flash chromatography using mixtures of dichloromethane/methanol as eluent with 45-60% of yield.

General Procedure C: Boc Removal (12-15, 20-23)

The N-Boc protected intermediates (1 mmol) were dissolved in a mixture of TFA/DCM (1/3, v/v), and triisopropylsilane (TIS, 0.25 mmol) was added. The mixtures were stirred at room temperature for 2 h. Then, a solution of NaOH (2 N) was added dropwise until pH 7 and diluted with dichloromethane. The organic layer was extracted, dried over Na_2SO_4, filtered, and concentrated in vacuo. The final products were obtained without further purification.

General procedure D: reductive amination (16-19)

Intermediates **7-10** (1.0 mmol) were dissolved in methanol under positive flux, added with a 37% aqueous solution of formaldehyde (1.5 eq) and stirred at room temperature overnight. Then, 3 eq of $NaBH_4$ were added and the mixture was reacted for further 1 h. Afterwards, the reaction was quenched by 10% aqueous solution of citric acid and the organic solvent was concentrated. Dichloromethane was added and the aqueous solution was extracted. The organic layer was dried over Na_2SO_4, filtered, and evaporated. The crude products were purified by flash chromatography using mixtures of DCM/MeOH as mobile phase furnishing the corresponding methylated derivatives with 45-55% of yields.

Synthesis of methyl 1-methyl-1H-indole-5-carboxylate (1)

Methyl indole-5-carboxylate (1.0 mmol) was dissolved in a mixture of DCM/DMF under magnetic stirring, and the temperature was set to 0 °C. To this solution, 1.5 mmol of NaH were added portionwise and the mixture was reacted for 30 min. Then, 1.5 equivalents of methyl iodide were added and the reaction was warmed to room temperature and maintained under stirring for further 12 h. After the completion of the reaction, the mixture was quenched by 10% aqueous solution of citric acid and diluted with dichloromethane. The organic phase was separated, dried over anhydrous Na_2SO_4, filtered, and evaporated in vacuo. Crude product was purified by column chromatography using n-hexane/ethyl acetate (1:1 v:v) as mobile phase, to obtain intermediate **1** as a whitish oil with 84% of yield. Rf: 0.45. ^{1}H NMR (400 MHz, CD_3OD) δ: 3.85 (s, 3H, CH_3); 6.58 (d, 1H, aryl, J= 4.0 Hz); 7.26 (d, 1H, aryl, J= 4.0 Hz); 7.43 (d, 1H, aryl, J= 8.0 Hz, aryl); 7.87 (d, 1H, aryl, J= 8.0 Hz, aryl); 8.33 (s, 1H, aryl). HR-MS m/z calcd for $C_{10}H_{10}NO_2$ [(M + H) $^+$]: 190.0863, found 190.0878.

Synthesis of 1-methyl-1H-indole-5-carboxylic acid (2)

To a solution of intermediate **1** (1 mmol) dissolved in methanol, 5 mL of NaOH 1M aqueous solution were added. The temperature was set at 100 ° C and the reaction mixture was stirred until the complete disappearance of the starting material, evidenced by TLC. The aqueous phase was quenched with a HCl 2M solution, diluted with ethyl acetate and separated. Then the organic layer was dried over anhydrous Na_2SO_4, filtered, and evaporated under vacuum affording intermediate **2** as a white powder without further purification. (94% yield). ^{1}H NMR (400 MHz, CD_3OD) δ: 3.85 (s, 3H, C*H3*); 6.58 (d, 1H, aryl, *J*= 4.0 Hz); 7.26 (d, 1H, aryl, *J*= 4.0 Hz); 7.43 (d, 1H, aryl, *J*= 8.0 Hz, aryl); 7.87 (d, 1H, aryl, *J*= 8.0 Hz, aryl); 8.33 (s, 1H, aryl). HR-MS m/z calcd for $C_{10}H_{10}NO_2$ [(M + H) $^+$]: 176.0706, found 176.0712.

tert-butyl 4-(2-(1-methyl-1H-indole-5-carboxamido)ethyl)piperazine-1-carboxylate (3)

Synthesized from **2** and tert-butyl 4-(2-aminoethyl)piperazine-1-carboxylate as an off-white oil in 70% yield using the general procedure A.

FC in dichloromethane/methanol 9/1, R*f*: 0.39. ^{1}H NMR ($CDCl_3$, 400 MHz) δ: 1.50 (s, 9H, C*H3*); 2.59-2.73 (m, 4H, C*H2*); 3.18 (m, 2H, C*H2*); 3.49 (s, 3H, C*H3*); 3.60-3.70 (m, 4H, C*H2*); 3.81 (m, 2H, C*H2*); 6.58 (d, 1H, aryl, *J*= 8.5 Hz); 7.26 (d, 1H, aryl, *J*= 7.8 Hz); 7.43 (d, 1H, aryl, *J*= 8.1 Hz); 7.73 (d, 1H, aryl, *J*= 8.0 Hz); 8.16 (s, 1H, aryl); HR-MS *m/z*: calcd for $C_{23}H_{28}N_3O_3$ [(M+H)$^+$]: 393.2052; found 393.2058.

tert-butyl (3-(1-methyl-1H-indole-5-carboxamido)propyl)carbamate (4)

Synthesized from **2** and tert-butyl (3-aminopropyl)carbamate as an off-white oil in 62% yield using the general procedure A.

FC in n-hexane/ethyl acetate 3:7, R*f*: 0.45. ^{1}H NMR ($CDCl_3$, 400 MHz) δ: 1.44 (s, 9H, C*H3*); 1.75-1.81 (m, 2H, C*H2*); 3.16 (t, 2H, C*H2*, *J*= 6.6 Hz); 3.43-3.48 (m, 2H, C*H2*); 3.76 (s, 3H, C*H3*); 6.51 (d, 1H, aryl, *J*= 3.7 Hz); 7.20 (d, 1H, aryl, *J*= 3.1 Hz); 7.37 (d, 1H, aryl, *J*= 8.6 Hz); 7.68 (d, 1H, aryl, *J*= 6.9 Hz); 8.12 (s, 1H, aryl); 8.27 (bs, 1H, N*H*); HR-MS *m/z*: calcd for $C_{18}H_{26}N_3O_3$ [(M+H)$^+$]: 331.1896; found 331.1901.

N-(3-(aminomethyl)benzyl)-1-methyl-1H-indole-5-carboxamide (5)

Synthesized from **2** and tert-butyl (3-(aminomethyl)benzyl)carbamate as an off-white oil in 65% yield using the general procedure A.

FC in n-hexane/ethyl acetate 1/1, R*f*: 0.46. ^{1}H NMR ($CDCl_3$, 400 MHz) δ: 1.44 (s, 9H, 3 C*H3*); 3.75 (s, 2H, C*H2*); 4.22 (d, 2H, C*H2*, *J*= 4.8 Hz); 4.57 (d, 2H, C*H2*, *J*= 5.6 Hz); 5.13 (bs, 1H, N*H*); 6.49 (d, 1H, aryl, *J*= 3.8 Hz); 6.98 (bs, 1H, CON*H*); 7.06 (d, 1H, aryl, *J*= 3.2 Hz); 7.15-7.28 (m, 5H, aryl); 7.73 (d, 1H, aryl, *J*= 10.3); 8.14 (s, 1H, aryl); HR-MS *m/z*: calcd for $C_{18}H_{20}N_3O$ [(M+H)$^+$]: 293.1528; found 293.1534.

1-methyl-N-(p-tolyl)-1H-indole-5-carboxamide (6)

Synthesized from **2** and p-toluidine as an off-white oil in 68% yield using the general procedure A. FC in dichloromethane/ethyl acetate 9.5/0.5. ^{1}H NMR ($CDCl_3$, 400 MHz) δ: 2.36 (s, 3H, C*H₃*); 3.80 (s, 3H, C*H₃*); 6.56 (d, 1H, aryl, *J*= 3.6 Hz); 7.12 (d, 1H, aryl, *J*= 3.2 Hz); 7.18 (d, 2H, aryl, *J*= 8.2 Hz); 7.32 (d, 1H, aryl, *J*= 8.6 Hz); 7.58 (d, 2H, aryl, *J*= 8.4 Hz); 7.78 (d, 1H, aryl, *J*= 8.6 Hz); 8.05 (bs, 1H, CON*H*); 8.19 (s, 1H, aryl); HR-MS *m/z*: calcd for $C_{17}H_{17}N_2O$ [(M+H)$^+$]: 264.1263; found 264.1268.

tert-butyl 4-(2-(3-(((4-hydroxybenzyl)amino)methyl)-1-methyl-1H-indole-5-carboxamido)ethyl)piperazine-1-carboxylate (7)

Synthesized from **3** as an off-white oil in 55% yield using the general procedure B.

FC in dichloromethane/methanol 9/1, R*f*: 0.39. ^{1}H NMR (CD_3OD, 400 MHz) δ: 1.46 (s, 9H, CH_3); 2.50 (t, 4H, 2 CH_2, *J*= 4.9 Hz); 2.63 (t, 2H, CH_2, *J*= 6.7 Hz); 3.41 (bs, 4H, 2 CH_2); 3.57 (t, 2H, CH_2, *J*=6.6 Hz); 3.81 (s, 3H, CH_3); 4.01 (s, 2H, CH_2); 6.79 (d, 1H, aryl, *J*= 8.4 Hz); 7.22 (d, 2H, aryl, *J*= 8.4 Hz); 7.30 (s, 1H, aryl); 7.42 (d, 2H, aryl, *J*= 8.7 Hz); 7.68 (d, 1H, aryl, *J*= 8.6 Hz); 8.06 (s, 1H, aryl); HR-MS *m/z*: calcd for $C_{29}H_{40}N_5O_4$ [(M+H)$^+$]: 522.3075; found 522.3079.

tert-butyl (3-(3-(((4-hydroxybenzyl)(methyl)amino)methyl)-1-methyl-1H-indole-5-carboxamido)propyl)carbamate (8)

Synthesized from **4** as an off-white oil in 52% yield using the general procedure D.

FC in dichloromethane/methanol 9:1, R*f*: 0.45. ^{1}H NMR (CD_3OD, 400 MHz) δ: 1.45 (s, 9H, CH_3); 1.78-1.83 (m, 2H, CH_2); 2.20 (s, 3H, CH_3); 3.18 (t, 2H, CH_2, *J*= 6.6 Hz); 3.46-3.49 (m, 4H, CH_2); 3.75 (s, 2H, CH_2); 3.81 (s, 3H, CH_3); 4.01 (s, 2H, CH_2); 6.76 (d, 2H, aryl, *J*= 8.5 Hz); 7.15 (d, 2H, aryl, *J*= 8.6 Hz); 7.23 (s, 1H, aryl); 7.41 (d, 1H, aryl, *J*= 8.6 Hz); 7.71 (d, 1H, aryl, *J*= 8.6 Hz); 8.18 (s, 1H, aryl); HR-MS *m/z*: calcd for $C_{27}H_{37}N_4O_4$ [(M+H)$^+$]: 481.2809; found 481.2811.

tert-butyl (3-((3-(((4-hydroxybenzyl)amino)methyl)-1-methyl-1H-indole-5-carboxamido)methyl)benzyl)carbamate (9)

Synthesized from **5** as a whitish oil in 51% yield using the general procedure B.

FC in dichloromethane/methanol 9/1, R*f*: 0.38. ^{1}H NMR (CD_3OD, 400 MHz) δ: 1.41 (s, 9H, CH_3); 3.72 (s, 2H, CH_2); 3.82 (s, 3H, CH_3); 3.96 (s, 2H, CH_2); 4.24 (s, 2H, CH_2); 4.62 (s, 2H, CH_2); 6.75 (d, 2H, aryl, *J*= 8.5 Hz); 7.14-7.19 (m, 3H, aryl); 7.24 (s, 1H, aryl); 7.30 (d, 2H, aryl, *J*= 7.3 Hz); 7.42 (d, 1H, aryl, *J*= 8.6 Hz); 7.76 (d, 1H, aryl, *J*= 10.2 Hz); 8.20 (s, 1H, aryl); HR-MS *m/z*: calcd for $C_{31}H_{37}N_4O_4$ [(M+H)$^+$]: 529.2809; found 529.2812.

tert-butyl (3-(3-(((3-hydroxybenzyl)amino)methyl)-1-methyl-1H-indole-5-carboxamido)propyl)carbamate (10)

Synthesized from **4** as an off-white oil in 45% yield using the general procedure B.

FC in dichloromethane/methanol 9/1, R*f*: 0.42. ^{1}H NMR (CD_3OD, 400 MHz) δ: 1.45 (s, 9H, C*H3*); 1.77-1.84 (m, 2H, C*H2*); 3.18 (t, 2H, C*H2*, *J*= 6.7 Hz); 3.47 (t, 2H, C*H2*, *J*= 6.7 Hz); 3.85 (s, 3H, C*H3*); 4.04 (s, 2H, C*H2*); 4.29 (s, 2H, C*H2*); 6.82 (d, 1H, aryl, *J*= 6.4 Hz); 6.88-6.91 (m, 2H, aryl); 7.23 (t, 1H, aryl, *J*= 7.8 Hz); 7.43 (s, 1H, aryl); 7.48 (d, 1H, aryl, *J*= 8.7 Hz); 7.75 (d, 1H, aryl, *J*= 7.0 Hz); 8.23 (s, 1H, aryl); HR-MS *m/z*: calcd for $C_{26}H_{35}N_4O_4$ [(M+H)$^+$]: 466.2580; found 466.2586.

3-(((3,4-dihydroxybenzyl)amino)methyl)-1-methyl-N-(p-tolyl)-1H-indole-5-carboxamide (11)

Synthesized from **6** as an off-white oil in 44% yield using the general procedure D.

FC in ethyl acetate/methanol 7/3, R*f*: 0.39. ^{1}H NMR (CD_3OD, 400 MHz) δ: 2.36 (s, 3H, C*H3*); 3.80 (s, 2H, C*H2*); 3.87 (s, 3H, C*H3*); 4.10 (s, 2H, C*H2*); 6.71-6.78 (m, 2H, aryl); 6.83 (d, 1H, aryl, *J*= 1.9 Hz); 7.20 (d, 2H, aryl, *J*= 8.2 Hz); 7.34 (s, 1H, aryl); 7.50 (d, 1H, aryl, *J*= 8.6 Hz); 7.58 (d, 2H, aryl, *J*= 8.4 Hz); 7.83 (d, 1H, aryl, *J*= 10.3 Hz); 8.30 (s, 1H, aryl). ^{13}C NMR (CD_3OD, 100 MHz) : 19.5; 31.6; 42.1; 51.8; 109.1; 115.0; 115.7; 118.7; 120.0; 121.0; 125.6; 127.0; 128.9; 130.0; 133.7; 136.2; 139.0; 144.7; 145.2; 168.6. HR-MS *m/z*: calcd for $C_{25}H_{26}N_3O_3$ [(M+H)$^+$]: 416.1969; found 416.1973.

3-(((4-hydroxybenzyl) amino)methyl)-1-methyl-N-(2-(piperazin-1-yl)ethyl)-1H-indole-5-carboxamide (12)

Synthesized from **7** as an off-white powder in 53% yield using the general procedure C.

Precipitated from DCM/diethyl ether. ^{1}H NMR (400 MHz, CD_3OD) δ: 2.89 (t, 2H, C*H2, J*= 6.3 Hz); 3.00 (bs, 4H, C*H2*); 3.31-3.33 (m, 4H, C*H2*); 3.66 (t, 2H, C*H2, J*= 6.3 Hz); 3.90 (s, 3H, C*H3*); 4.17 (s, 2H, C*H2*); 4.44 (s, 2H, C*H2*); 6.86 (d, 2H, aryl*, J*= 8.5 Hz); 7.31 (d, 2H, aryl, *J*= 8.5 Hz); 7.53-7.55 (m, 2H, aryl); 7.78 (d, 1H, aryl*, J*= 8.7 Hz); 8.25 (s, 1H, aryl). ^{13}C NMR (100 MHz, CD_3OD) δ: 31.9, 36.3, 40.8, 43.0, 49.3, 49.9, 56.7, 105.1, 109.5, 115.5, 118.3, 121.1, 121.6, 125.9, 126.8, 131.2, 132.6, 138.9, 158.5, 170.1. HR-MS m/z calcd for $C_{24}H_{32}N_5O_2$ [(M + H)]$^+$: 422.2551; found 422.2955.

N-(3-aminopropyl)-3-(((4-hydroxybenzyl) amino)methyl)-1-methyl-1H-indole-5-carboxamide (13)

Obtained from **8** as an off-white powder in 51% yield using the general procedure C.

Precipitated from DCM/diethyl ether. ^{1}H NMR (400 MHz, CD_3OD) δ: 1.98-2.05 (m, 2H, C*H2,*); 3.05 (t, 2H, C*H2, J*= 7.2 Hz); 3.57 (t, 2H, C*H2, J*= 6.5 Hz); 3.89 (s, 3H, C*H3*); 4.17 (s, 2H, C*H2*); 4.44 (s, 2H, C*H2*); 6.85 (d, 2H, aryl*, J*= 8.5 Hz); 7.31 (d, 2H, aryl, *J*= 8.5 Hz); 7.53-7.55 (m, 2H, aryl); 7.79 (d, 1H, aryl*, J*= 8.7 Hz); 8.27 (s, 1H, aryl). ^{13}C NMR (100 MHz, CD_3OD) δ: 27.6, 31.9, 36.1, 36.9, 40.8, 49.9, 105.1, 109.6, 115.5, 118.4, 121.1, 121.6, 125.6, 126.8, 131.2, 132.6, 138.9, 158.5, 170.5. HR-MS m/z calcd for $C_{21}H_{27}N_4O_2$ [(M + H)$^+$]: 367.2129; found 367.2132.

N-(3-(aminomethyl)benzyl)-3-(((4-hydroxybenzyl)amino)methyl)-1-methyl-1H-indole-5-carboxamide (14)

Synthesized from **9** as an off-white powder in 45% yield using the general procedure C.

Precipitated from DCM/diethyl ether. ^{1}H NMR (CD_3OD, 400 MHz) δ: 3.90 (s, 3H, CH_3); 4.13 (s, 2H, CH_2); 4.17 (s, 2H, CH_2); 4.45 (s, 2H, CH_2); 4.67 (s, 2H, CH_2); 6.85 (d, 2H, aryl, *J*= 8.6 Hz); 7.31 (d, 2H, aryl, *J*= 8.6 Hz); 7.37 (d, 1H, aryl, *J*= 7.2 Hz); 7.43-7.56 (m, 4H, aryl); 7.83 (dd, 1H, aryl, *J'*= 1.6, *J''*= 8.7 Hz); 8.30 (d, 1H, *J*= 1.2 Hz). ^{13}C NMR (CD_3OD, 100 MHz) : 31.9; 40.8; 42.9; 43.0; 49.9; 105.1; 109.6; 115.5; 118.4; 121. 6; 125.9; 126.8; 127.3; 127.8; 127.9; 129.0; 131.2; 132.6; 133.3; 138.9; 140.4; 158.5; 169.8; HR-MS *m/z*: calcd for $C_{26}H_{29}N_4O_2$ [(M+H)$^+$]: 429.2285; found 429.2290.

N-(3-aminopropyl)-3-(((3-hydroxybenzyl)amino)methyl)-1-methyl-1H-indole-5-carboxamide (15)

Synthesized from **10** as a yellowish powder in 47% yield using the general procedure C.

Precipitated from DCM/diethyl ether. ^{1}H NMR (CD_3OD, 400 MHz) δ: 1.99-2.05 (m, 2H, CH_2); 3.05 (t, 2H, CH_2, *J*= 7.1 Hz); 3.57 (t, 2H, CH_2, *J*= 6.4 Hz); 3.91 (s, 3H, CH_3); 3.67 (t, 2H, CH_2, *J*= 6.2 Hz); 4.20 (s, 2H, CH_2); 4.48 (s, 2H, CH_2); 6.87-6.95 (m, 3H, aryl); 7.28 (t, 1H, aryl, *J*= 7.8 Hz); 7.55 (s, 1H, aryl); 7.56 (s, 1H, aryl); 7.79 (d, 1H, aryl, *J*= 8.7 Hz). ^{13}C NMR (CD_3OD, 100 MHz) : 27.6; 31.9; 36.1; 36.9; 41.1; 50.1; 104.9; 109.6; 116.1; 116.3; 118.4; 120.3; 121.1; 125.7; 126.8; 130.0; 132.5; 132.7; 138.9; 158.0; 170.5; HR-MS *m/z*: calcd for $C_{21}H_{27}N_4O_2$ [(M+H)$^+$]: 367.2129; found 367.2133.

tert-butyl 4-(2-(3-(((4-hydroxybenzyl)(methyl)amino)methyl)-1-methyl-1H-indole-5-carboxamido)ethyl)piperazine-1-carboxylate (16)

Synthesized from **12** as an off-white oil in 40% yield using the general procedure D.

FC in dichloromethane/methanol 9/1, R*f*: 0.39. ^{1}H NMR (CD_3OD, 400 MHz) δ: 1.47 (s, 9H, CH_3); 2.27 (s, 3H, CH_3); 2.54 (t, 4H, CH_2, *J*= 5.0 Hz); 2.68 (t, 2H, CH_2, *J*= 6.7 Hz); 3.46 (t, 4H, CH_2, *J*= 4.6 Hz); 3.57-3.66 (m, 4H, CH_2); 3.84 (s, 2H, CH_2); 3.85 (s, 3H, CH_3); 6.78 (d, 1H, aryl, *J*= 8.4 Hz); 7.17 (d, 2H, aryl, *J*= 8.5 Hz); 7.29 (s, 1H, aryl); 7.45 (d, 2H, aryl, *J*= 8.6 Hz); 7.71 (d, 1H, aryl, *J*= 8.6 Hz); 8.17 (s, 1H, aryl); HR-MS *m/z*: calcd for $C_{30}H_{42}N_5O_4$ [(M+H)$^+$]: 536.3231; found 536.3234.

tert-butyl (3-(3-(((4-hydroxybenzyl)(methyl)amino)methyl)-1-methyl-1H-indole-5-carboxamido)propyl)carbamate (17)

Synthesized from **13** as an off-white oil in 52% yield using the general procedure D.

FC in dichloromethane/methanol 9:1, R*f*: 0.45. ^{1}H NMR (CD_3OD, 400 MHz) δ: 1.45 (s, 9H, CH_3); 1.78-1.83 (m, 2H, CH_2); 2.20 (s, 3H, CH_3); 3.18 (t, 2H, CH_2, *J*= 6.6 Hz); 3.46-3.49 (m, 4H, CH_2); 3.75 (s, 2H, CH_2); 3.81 (s, 3H, CH_3); 4.01 (s, 2H, CH_2); 6.76 (d, 2H, aryl, *J*= 8.5 Hz); 7.15 (d, 2H, aryl, *J*= 8.6 Hz); 7.23 (s, 1H, aryl); 7.41 (d, 1H, aryl, *J*= 8.6 Hz); 7.71 (d, 1H, aryl, *J*= 8.6 Hz); 8.18 (s, 1H, aryl); HR-MS *m/z*: calcd for $C_{27}H_{37}N_4O_4$ [(M+H)$^+$]: 481.2809; found 481.2811.

tert-butyl (3-((3-(((4-hydroxybenzyl)(methyl)amino)methyl)-1-methyl-1H-indole-5-carboxamido)methyl)benzyl)carbamate (18)

Synthesized from **14** as a whitish oil in 44% yield using the general procedure D.

FC in dichloromethane/methanol 9/1, R*f*: 0.38. ^{1}H NMR (CD_3OD, 400 MHz) δ: 1.28 (s, 9H, C*H3*); 2.45 (s, 3H, C*H3*); 3.74 (s, 3H, C*H3*); 3.97 (s, 2H, CH2); 4.10 (s, 2H, C*H2*); 4.22 (s, 2H, C*H2*); 4.49 (s, 2H, C*H2*); 6.71 (d, 2H, aryl, *J*= 8.6 Hz); 7.05 (t, 1H, aryl, *J*= 3.5 Hz); 7.15-7.19 (m, 3H, aryl); 7.37 (d, 2H, aryl, *J*= 5.0 Hz); 7.68 (d, 2H, aryl, *J*= 8.7 Hz); 8.16 (s, 1H, aryl); HR-MS *m/z*: calcd for $C_{32}H_{39}N_4O_4$ [$(M+H)^+$]: 543.2966; found 543.2969.

tert-butyl (3-(3-(((3-hydroxybenzyl)(methyl)amino)methyl)-1-methyl-1H-indole-5-carboxamido)propyl)carbamate (19)

Synthesized from **15** as an off-white oil in 49% yield using the general procedure D.

FC in dichloromethane/methanol 9/1, R*f*: 0.42. ^{1}H NMR (CD_3OD, 400 MHz) δ: 1.45 (s, 9H, C*H3*); 1.77-1.83 (m, 2H, C*H2*); 2.25 (s, 3H, C*H3*); 3.18 (t, 2H, C*H2*, *J*= 6.7 Hz); 3.47 (t, 2H, C*H2*, *J*= 6.7 Hz); 3.54 (s, 2H, C*H2*); 3.80 (s, 2H, C*H2*); 3.82 (s, 3H, C*H3*); 6.72 (d, 1H, aryl, *J*= 6.4 Hz); 6.81-6.83 (m, 2H, aryl); 7.15 (t, 1H, aryl, *J*= 7.8 Hz); 7.25 (s, 1H, aryl); 7.41 (d, 1H, aryl, *J*= 8.6 Hz); 7.71(d, 1H, aryl, *J*=8.6 Hz); 8.20 (s, 1H, aryl); HR-MS *m/z*: calcd for $C_{27}H_{37}N_4O_4$ [$(M+H)^+$]: 480.2737; found 480.2744.

3-(((4-hydroxybenzyl)(methyl)amino)methyl)-1-methyl-N-(2-(piperazin-1-yl)ethyl)-1H-indole-5-carboxamide (20)

Derivative **20** was synthesized from **16** as an off-white powder in 53% yield using the general procedure C.

Precipitated from DCM/diethyl ether. ^{1}H NMR (400 MHz, CD_3OD) δ: 2.73 (s, 3H, C*H3*); 3.21 (t, 2H, C*H2*, *J*= 6.0 Hz); 3.38 (bs, 4H, C*H2*); 3.47-3.50 (m, 4H, C*H2*); 3.76 (t, 2H, C*H2*, *J*= 6.0 Hz); 3.92 (s, 3H, C*H3*); 4.16 (d, 1H, C*H*, *J*= 12.8 Hz); 4.48 (t, 2H, C*H2* *J*= 12.4 Hz); 4.66 (d, 1H, CH, *J*= 13.7 Hz); 6.89 (d, 2H, aryl, *J*= 8.5 Hz); 7.33 (d, 2H, aryl, *J*= 8.5 Hz); 7.56 (d, 1H, aryl, *J*= 8.7 Hz); 7.62 (s, 1H, aryl); 7.81 (d, 1H, aryl, *J*= 10.2 Hz); 8.24 (s, 1H, aryl). ^{13}C NMR (100 MHz, CD_3OD) δ: 32.0, 35.4, 37.5, 41.8, 49.0, 49.9, 56.8, 58.7, 103.2, 109.8, 115.7, 118.4, 120.0, 121.3. 126.0, 127.4, 132.3, 134.0, 139.0, 159.0, 170.3. HR-MS m/z calcd for $C_{25}H_{34}N_5O_2$ [$(M + H)^+$] : 436.2707; found 436.2711.

N-(3-aminopropyl)-3-(((4-hydroxybenzyl) (methyl)amino)methyl)-1-methyl-1H-indole-5 carboxamide (21)

Obtained from **17** as a white powder in 47% yield using the general procedure C.

Precipitated from DCM/diethyl ether. ^{1}H NMR (400 MHz, CD_3OD) δ: 1.98-2.05 (m, 2H, C*H2*); 2.73 (s, 3H, CH_3); 3.05 (t, 2H, C*H2*, *J*= 7.2 Hz); 3.56 (t, 2H, C*H2*, *J*= 6.6 Hz); 3.91 (s, 3H, C*H3*); 4.15 (d, 1H, C*H*, *J*= 12.9 Hz); 4.48 (t, 2H, C*H2*, *J*= 12.3 Hz); 4.66 (d, 1H, CH, *J*= 13.7 Hz); 6.89 (d, 2H, aryl,

J= 8.6 Hz); 7.33 (d, 2H, aryl, *J*= 8.6 Hz); 7.56 (d, 1H, aryl, *J*= 8.7 Hz); 7.61 (s, 1H, aryl); 7.81 (d, 1H, aryl, *J*= 8.7 Hz); 8.24 (s, 1H, aryl). ^{13}C NMR (100 MHz, CD_3OD) δ: 27.6, 32.0, 36.1, 36.9, 37.5, 49.9, 58.6, 103.2, 109.8, 115.7, 118.2, 120.0, 121.3, 126.1, 127.4, 132.3, 134.0, 138.9, 159.0, 170.3. HR-MS m/z calcd for $C_{22}H_{29}N_4O_2$ [(M + H)$^+$]: 381.2285; found 381.2288.

N-(3-(aminomethyl)benzyl)-3-(((4-hydroxybenzyl)(methyl)amino)methyl)-1-methyl-1H-indole-5-carboxamide (22)

Synthesized from **18** as an off-white powder in 42% yield using the general procedure C. Precipitated from DCM/diethyl ether. ^{1}H NMR (CD_3OD, 400 MHz) δ: 2.73 (s, 3H, C*H*3); 3.92 (s, 3H, C*H*3); 4.13 (s, 2H, C*H*2); 4.09-4.19 (m, 4H, C*H*2); 4.48 (t, 2H, C*H*2, *J*= 14.2 Hz); 4.67 (s, 2H, C*H*2); 6.89 (d, 2H, aryl, *J*= 8.8 Hz); 7.33 (d, 2H, aryl, *J*= 8.6 Hz); 7.37 (d, 1H, aryl, *J*= 7.3 Hz); 7.42-7.51(m, 3H, aryl); 7.57 (d, 1H, aryl, *J*= 8.7 Hz); 7.85 (dd, 1H, aryl, *J'*= 1.6, *J''*= 8.7 Hz); 8.29 (d, 1H, aryl, *J*= 1.2 Hz). ^{13}C NMR (CD_3OD, 100 MHz) δ: 32.0; 37.5; 42.9; 43.0; 50.0; 58.6; 103.1; 109.8; 115.7; 118.3; 120.0; 121. 4; 125.9; 126.4; 127.3; 127.4; 129.0; 132.3; 133.3; 134.0; 138.9; 140.3; 158.5; 169.6; HR-MS *m/z*: calcd for $C_{27}H_{31}N_4O_2$ [(M+H)$^+$]: 443.2442; found 443.2447.

N-(3-aminopropyl)-3-(((3-hydroxybenzyl)(methyl)amino)methyl)-1-methyl-1H-indole-5-carboxamide (23)

Synthesized from **19** as a yellowish powder in 48% yield using the general procedure C. Precipitated from DCM/diethyl ether. ^{1}H NMR (CD_3OD, 400 MHz) δ: 1.99-2.06 (m, 2H, C*H*2); 2.77 (s, 3H, C*H*3) 3.05 (t, 2H, C*H*2, *J*= 7.3 Hz); 3.57 (t, 2H, C*H*2, *J*= 6.6 Hz); 3.93 (s, 3H, C*H*3); 4.19-4.47 (m, 2H, C*H*2); 4.60 (s, 2H, C*H*2); 6.92-6.97 (m, 3H, aryl); 7.30-7.34 (m, 1H, aryl, *J*= 7.8 Hz); 7.58 (d, 1H, aryl, *J*= 8.7 Hz); 7.63 (s, 1H, aryl); 7.81 (dd, 1H, aryl, *J'*= 1.6 Hz, *J''*= 8.7 Hz); 8.26 (s, 1H, aryl); ^{13}C NMR (CD_3OD, 100 MHz) δ: 27.6; 32.1; 36.2; 38.0; 50.3; 58.8; 103.0; 109.8; 116.6; 117.3; 118.2; 121.3; 121.4; 126.1; 127.4; 130.2; 131.0; 134.1; 139.0; 158.1; 170.3; HR-MS *m/z*: calcd for $C_{22}H_{29}N_4O_2$ [(M+H)$^+$]: 381.2285; found 381.2291.

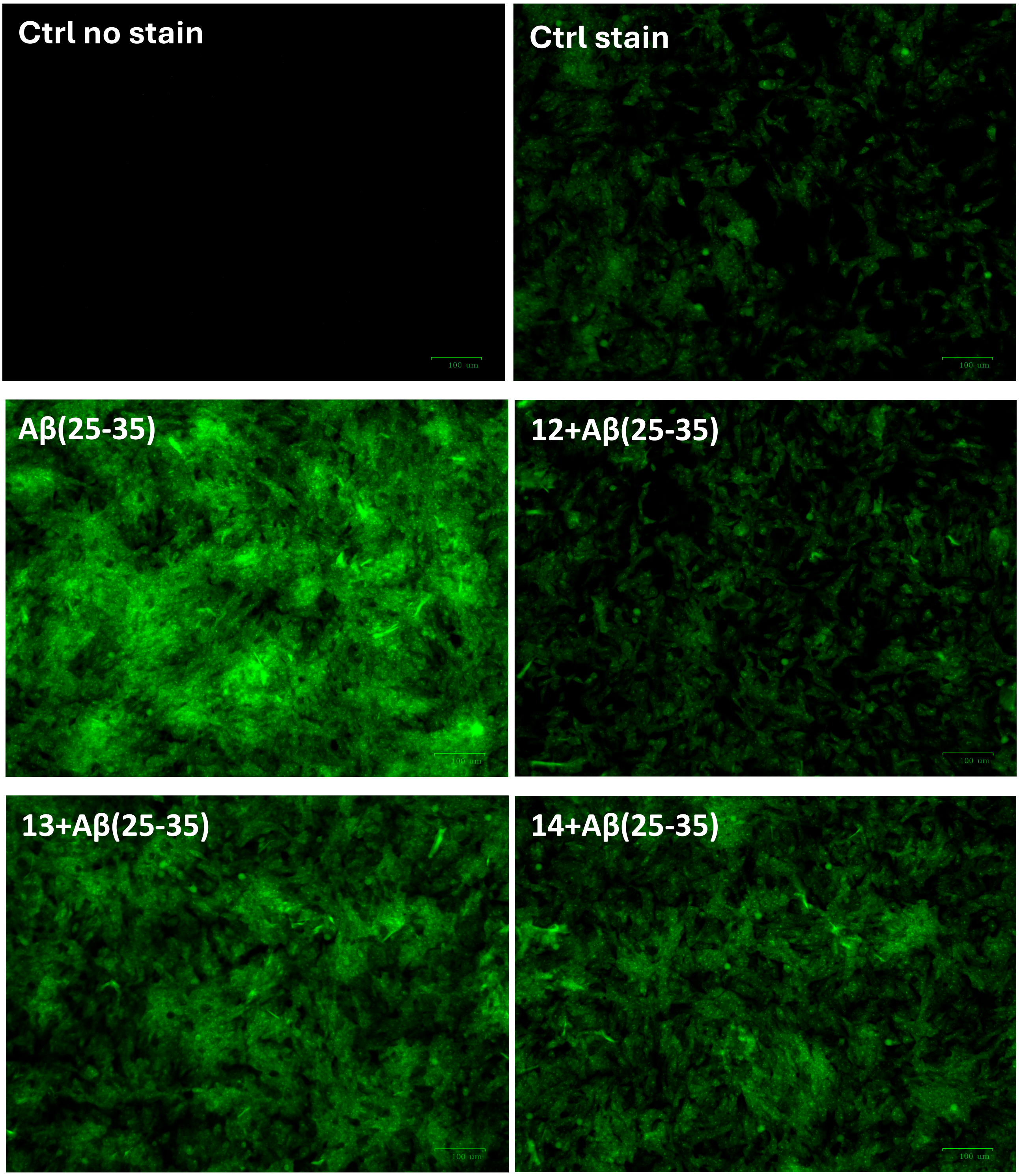
Ctrl no stain
Ctrl stain
Aβ(25-35)
12+Aβ(25-35)
13+Aβ(25-35)
14+Aβ(25-35)

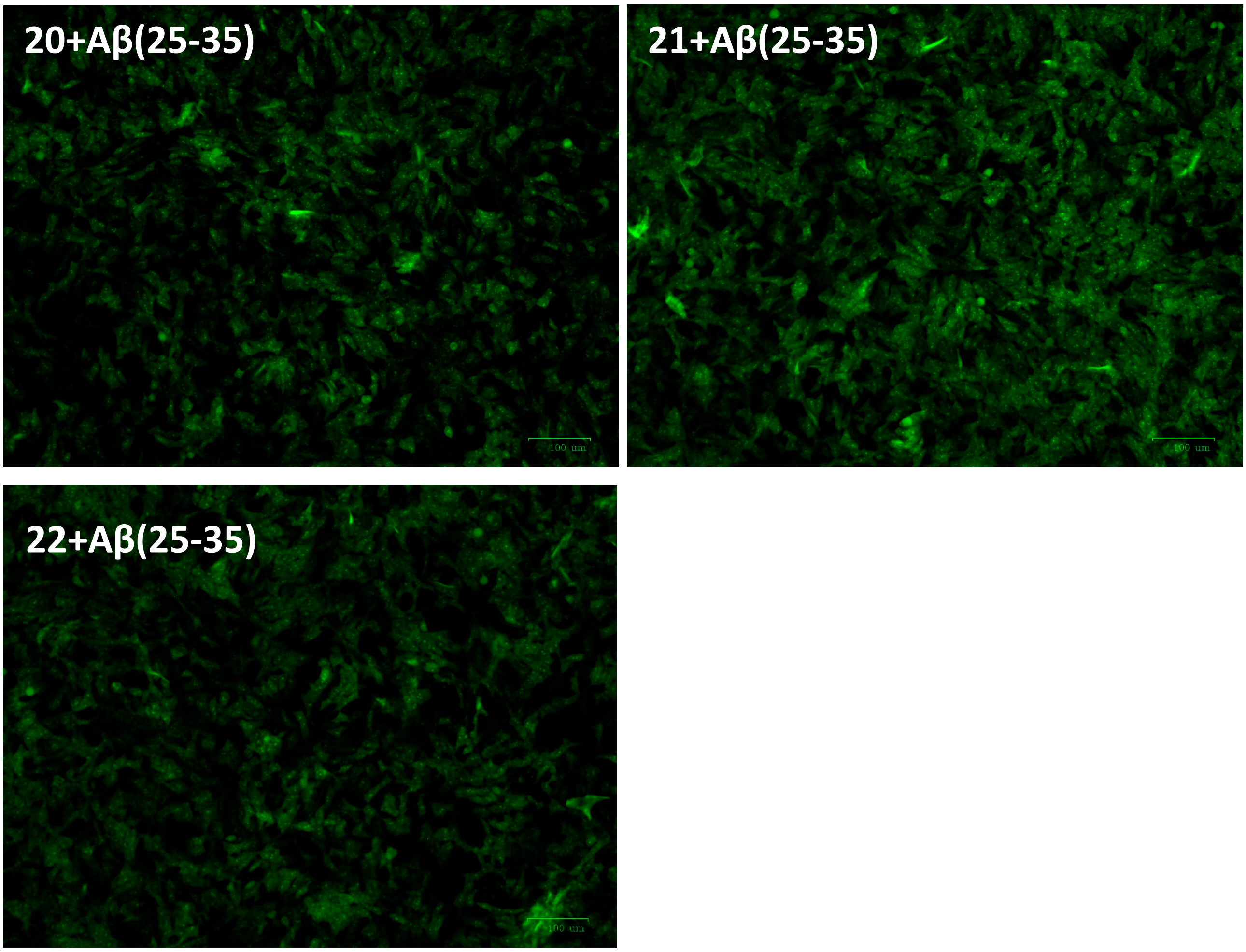

Figure S1. Thioflavin T fluorescence microscopy raw images of SH-SY5Y cells treated with 30 µM indole-based compounds and Aβ(25-35) 40 µM. Scale bar: 100 µm. (N ≥ 10). Cells were observed at 20× magnification.

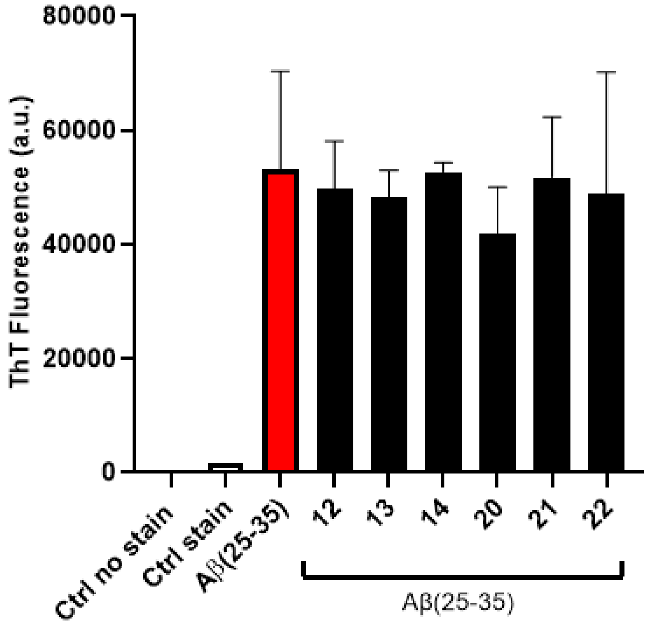

Figure S2. Thioflavin T fluorescence assay showing Aβ(25-35) 40 μM aggregation after co-incubation with test compounds (30 μM) for 24 hours. No significant reduction in fluorescence intensity was observed, indicating that these compounds did not inhibit aggregation of the amyloid peptide.

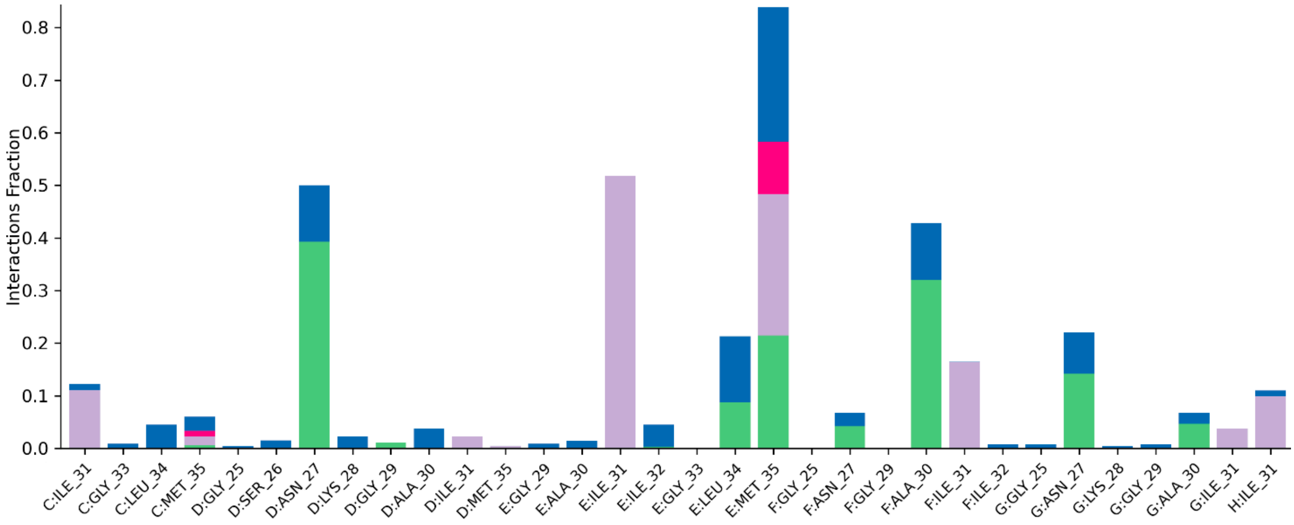

Figure S3. Contact histograms during the simulation of **20**-Aβ(25-35) model.

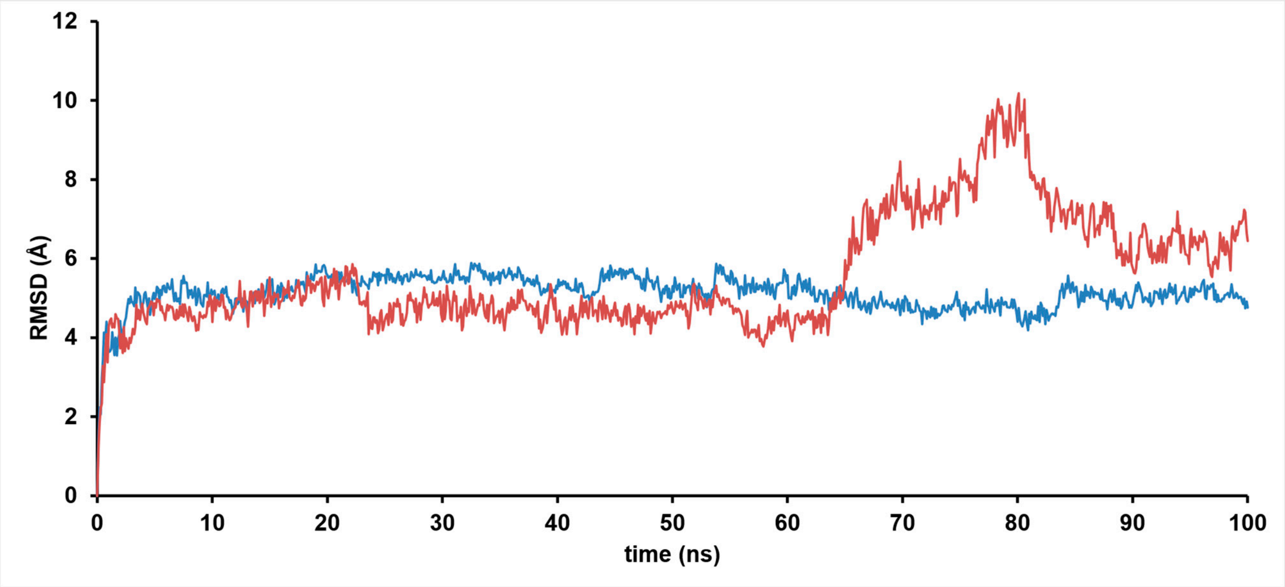

Figure S4. Backbone RMSD (Å) plot of Aβ(25-35) model in free (blue line), and bound to **20** (brown line) state from molecular dynamics simulations (simulation time = 100 ns, temperature = 310 K). The RSMD values focus on chain C-H, because monomers at the edge of β-sheet are mostly affected by the absence of other flanking strands.

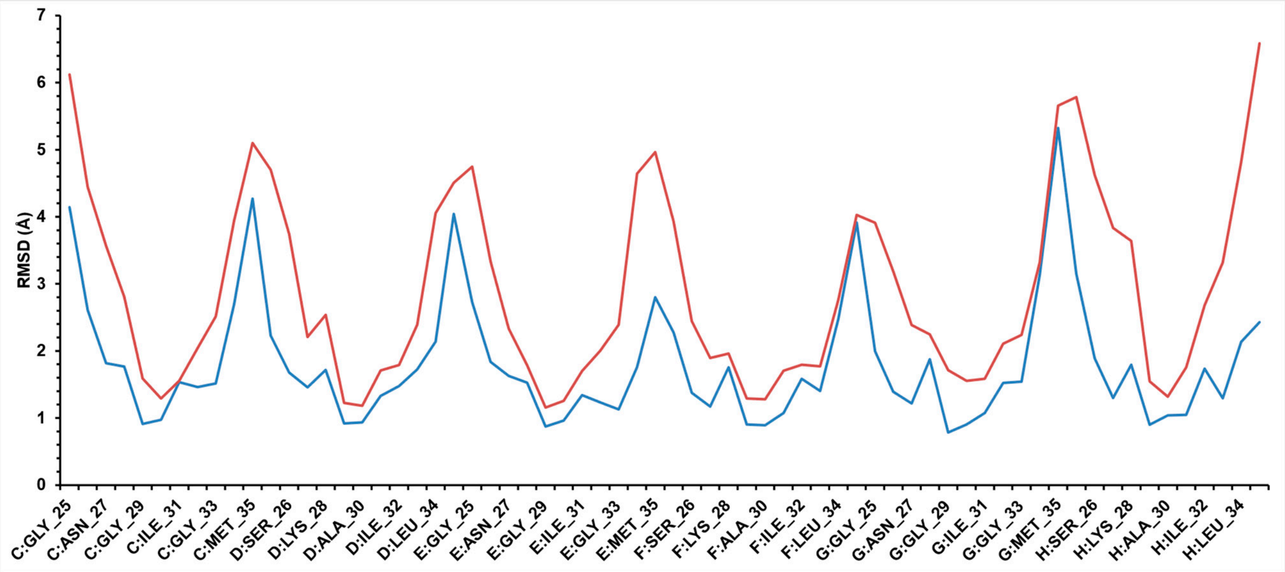

Figure S5. The Root Mean Square Fluctuation (RMSF) of side chains of Aβ(25-35) model in free (blue line), and bound to **20** (brown line) state as a function of simulation time (ns).

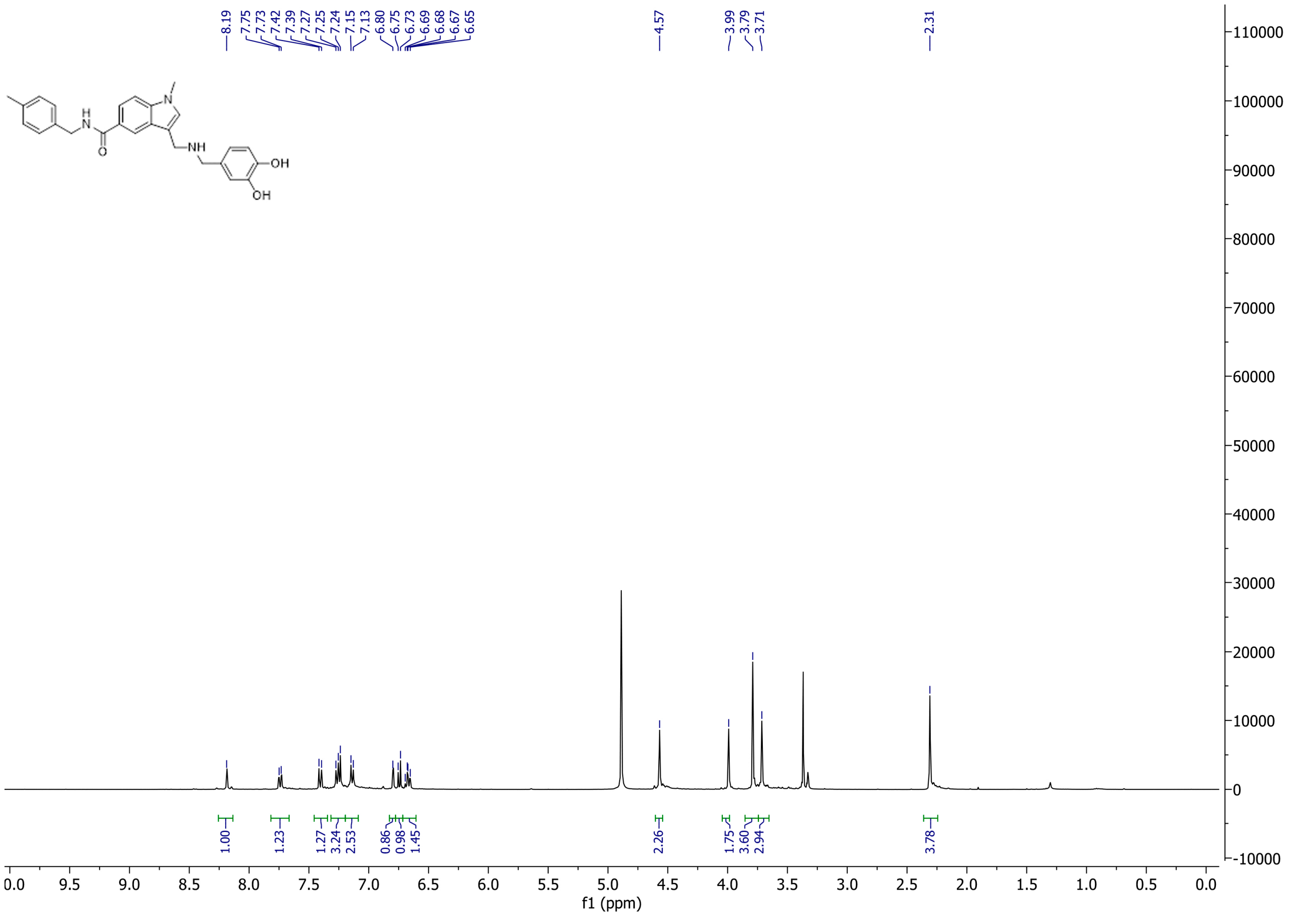

Figure S6: 1H NMR spectra of compound **11**

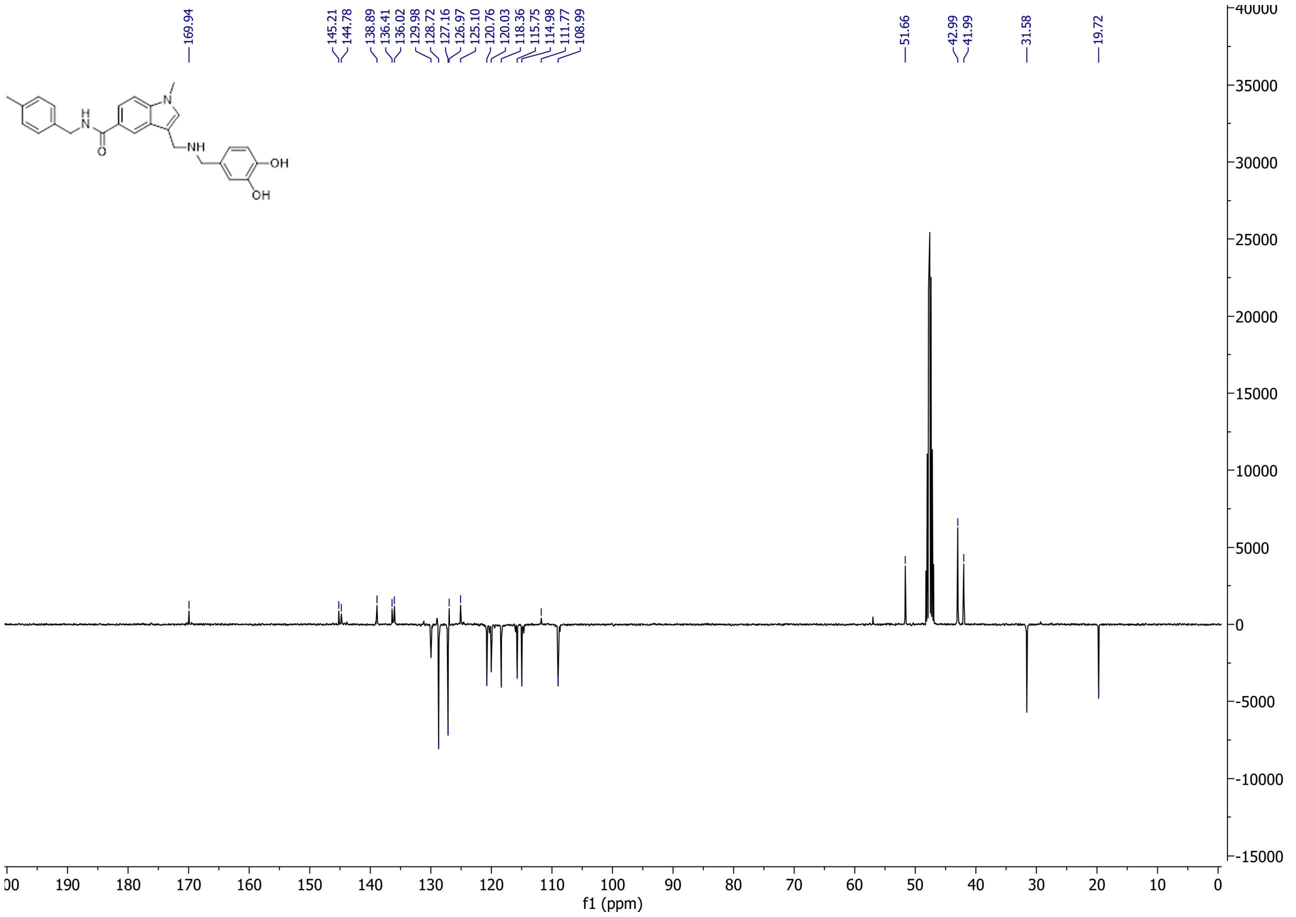

Figure S7: DEPT spectra of compound **11**

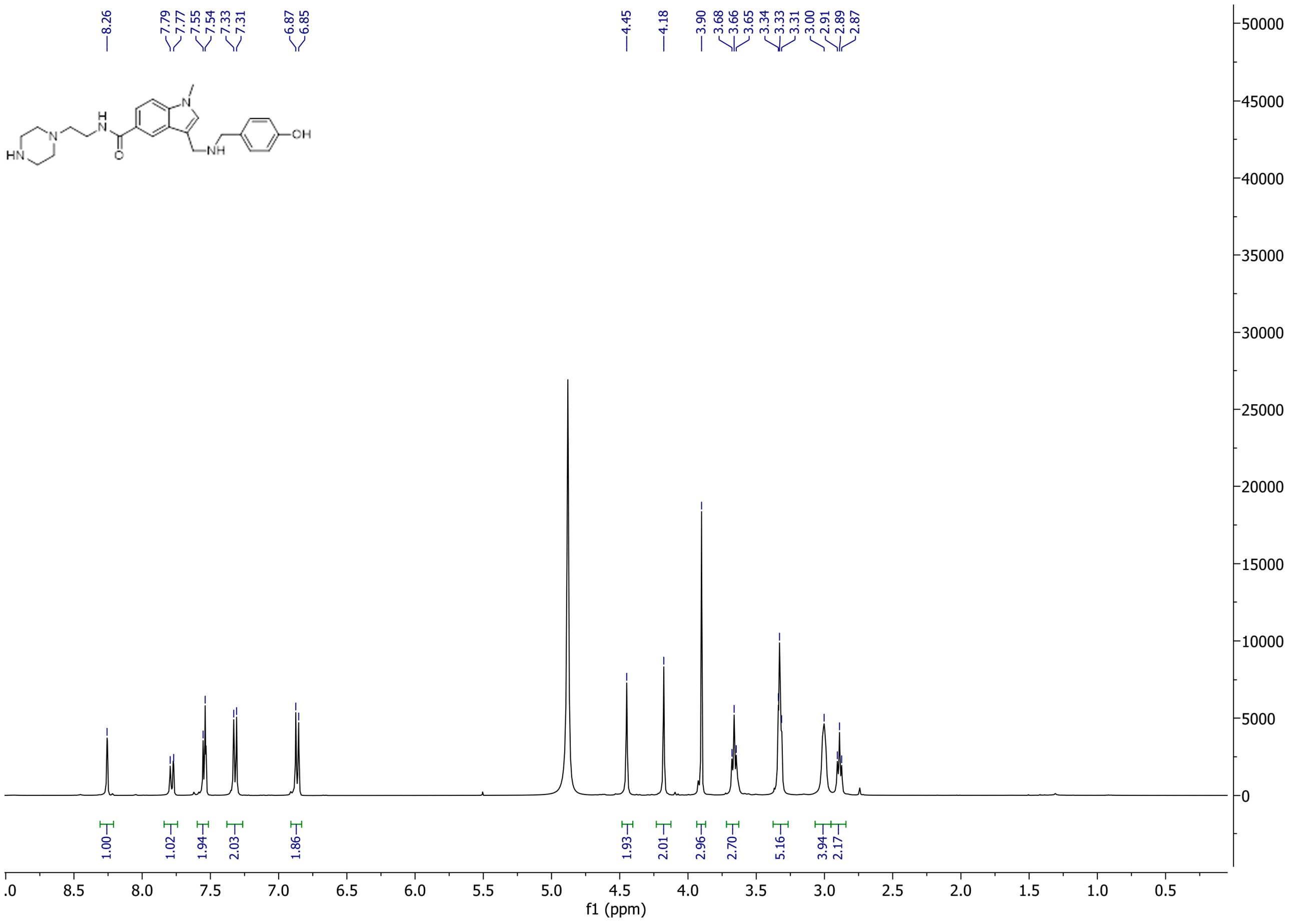

Figure S8: 1H NMR spectra of compound **12**

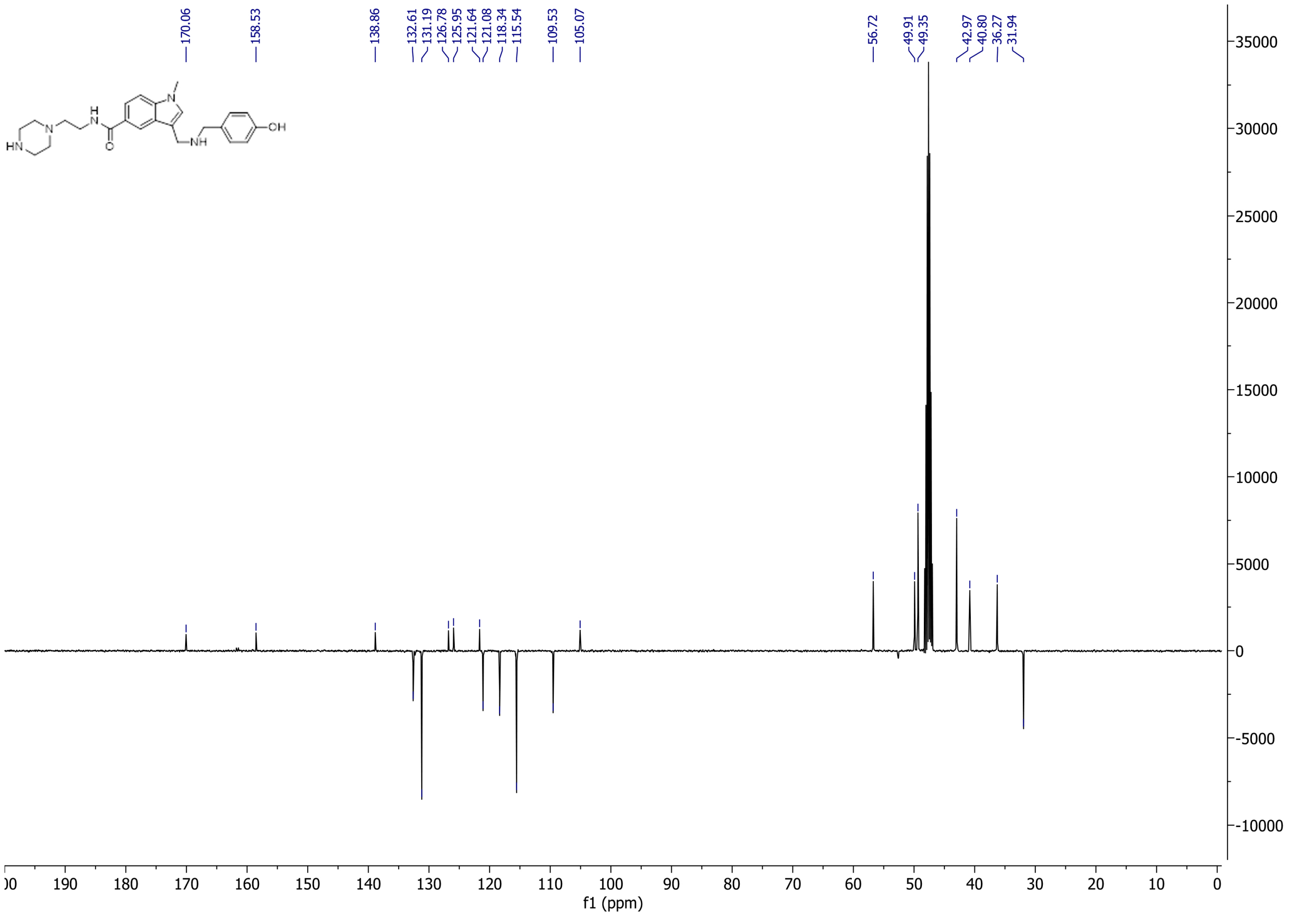

Figure S9: DEPT spectra of compound **12**

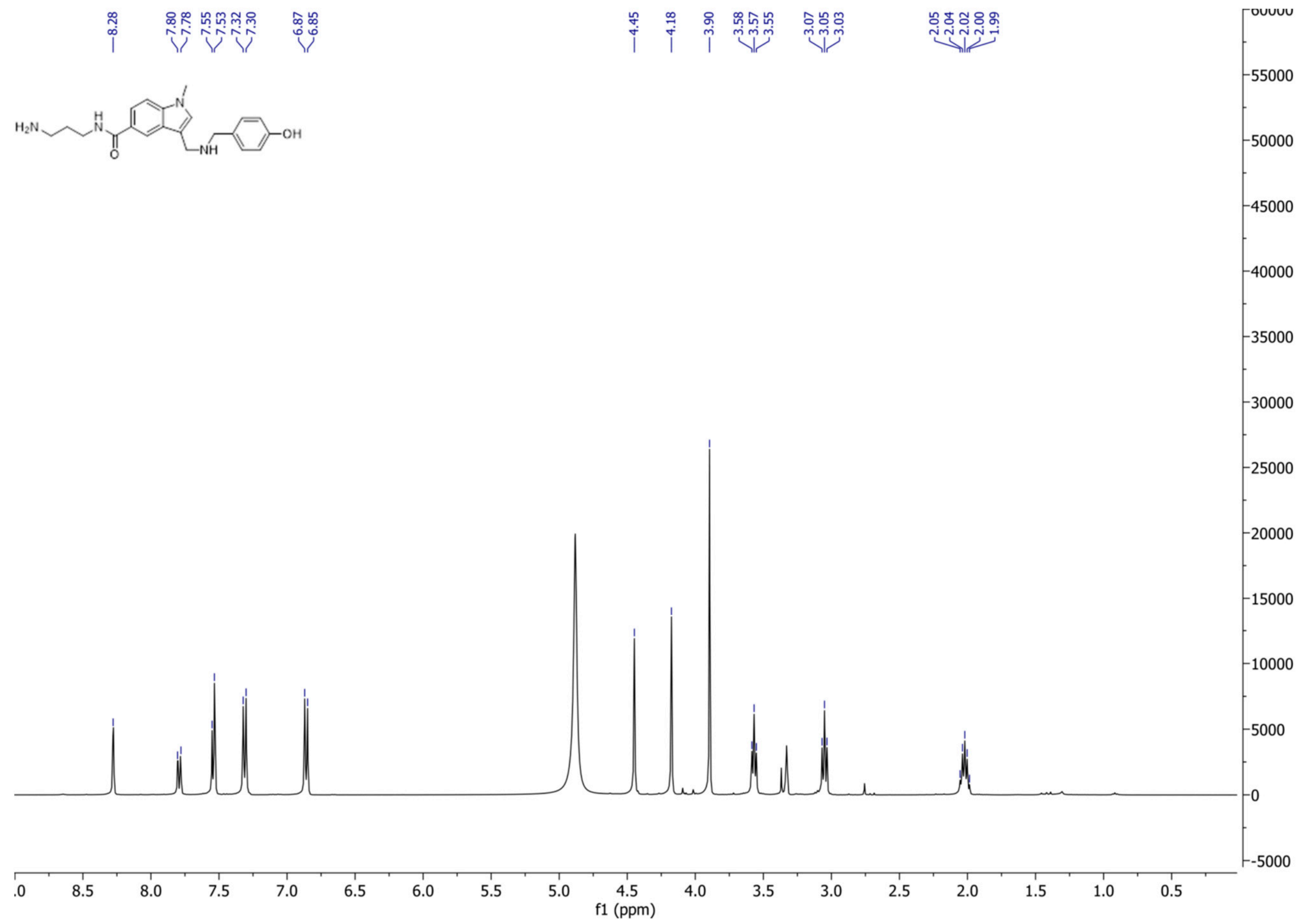

Figure S10: 1H NMR spectra of compound **13**

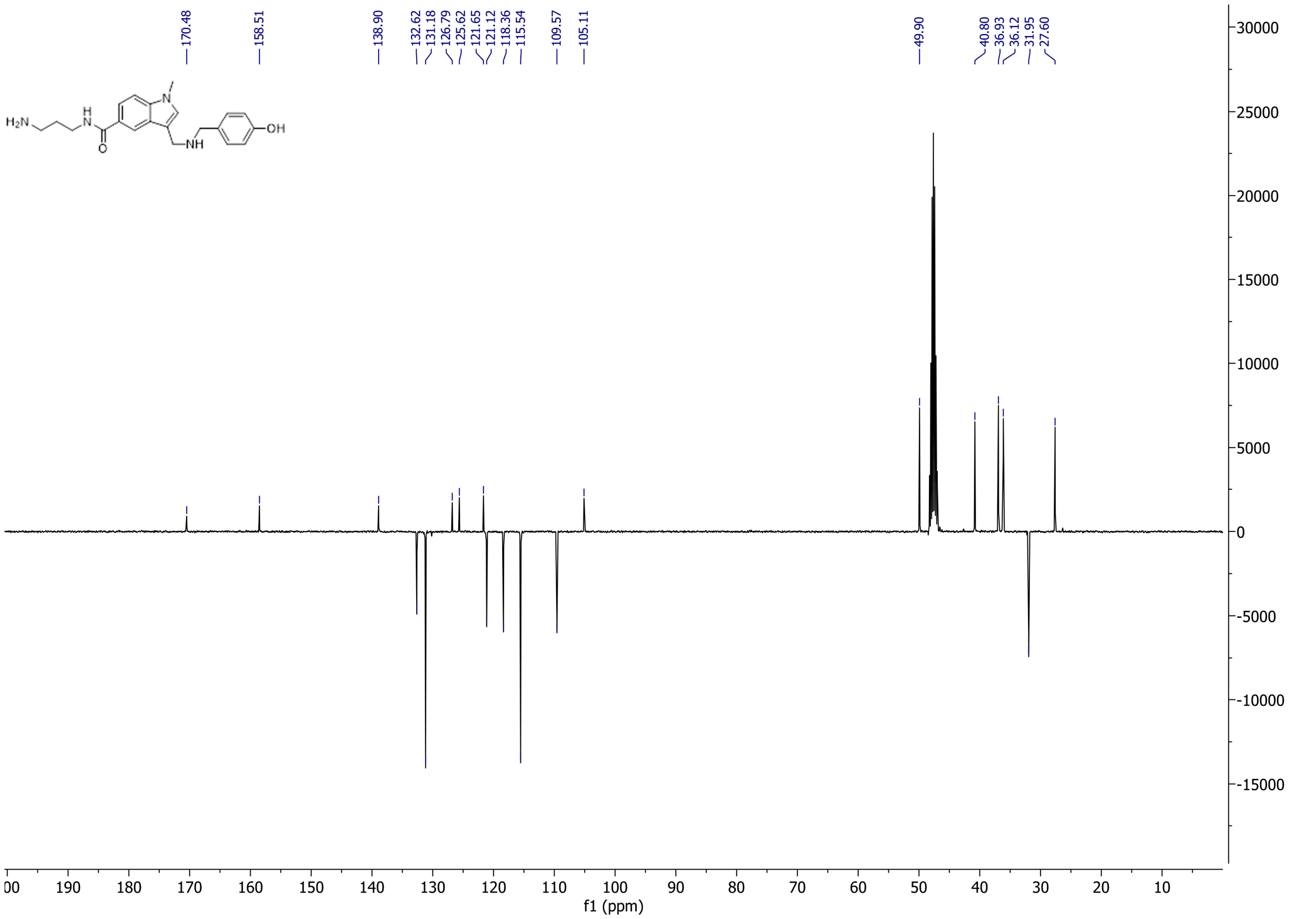

Figure S11: DEPT spectra of compound **13**

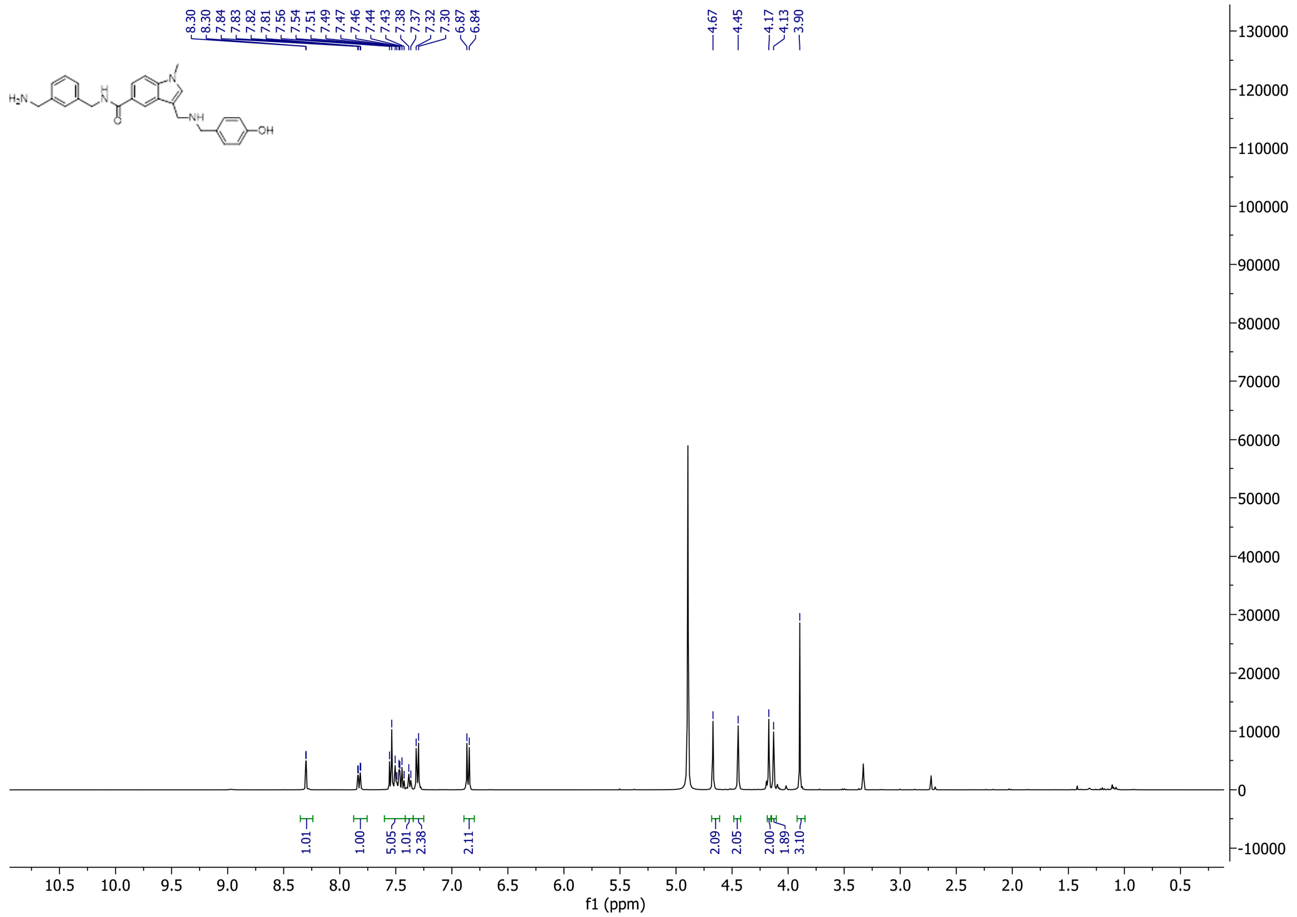

Figure S12: 1H NMR spectra of compound **14**

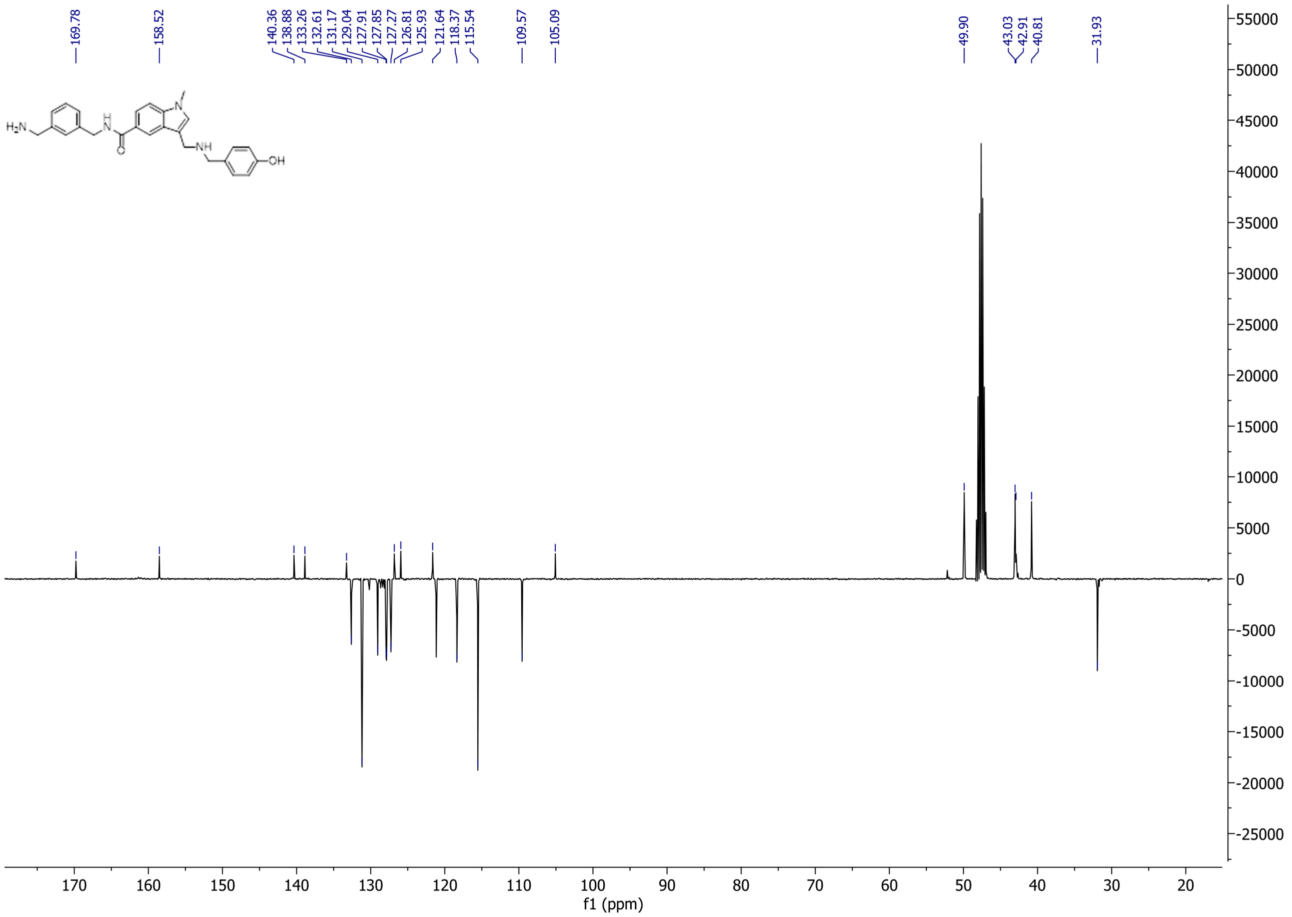

Figure S13: DEPT spectra of compound **14**

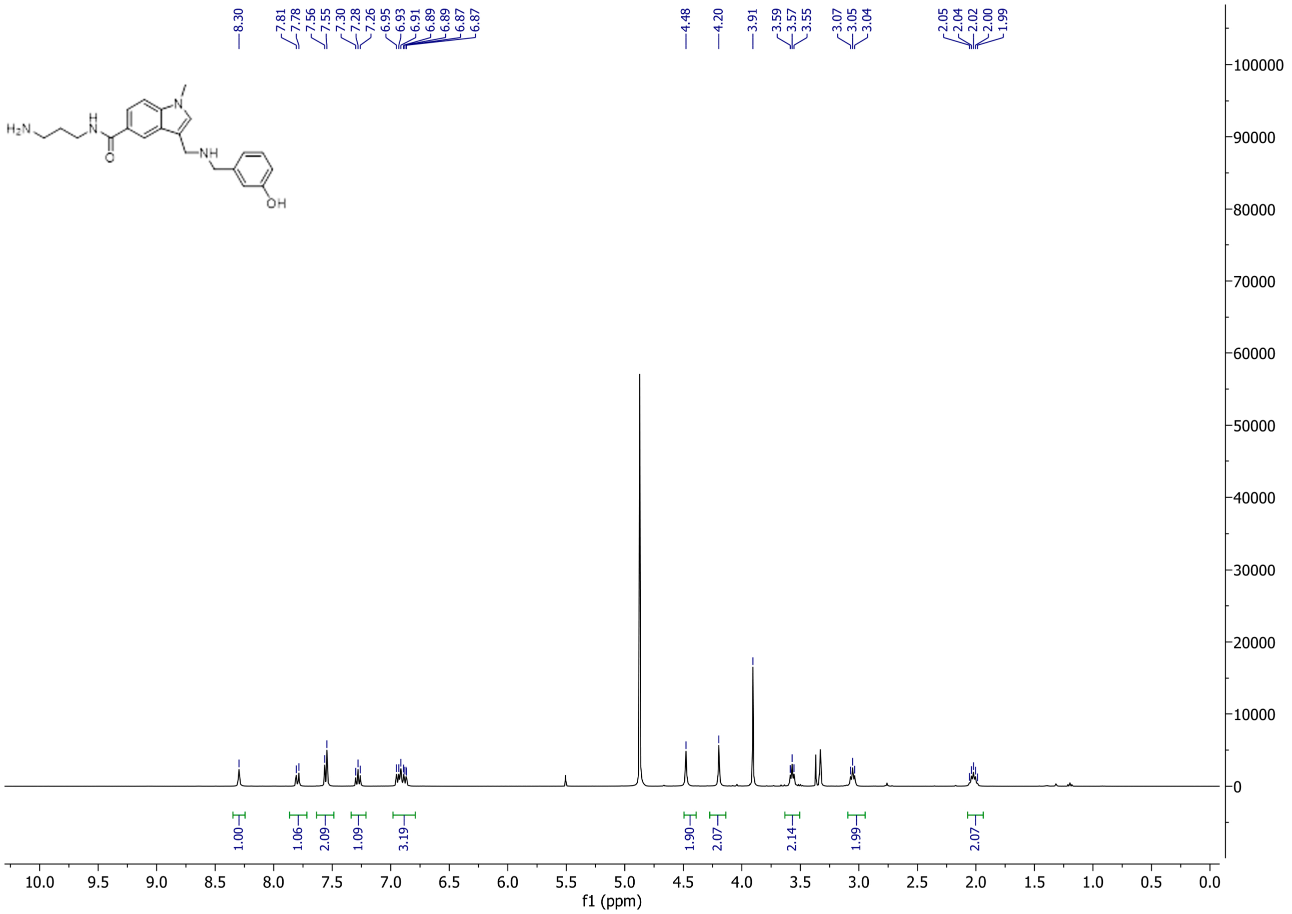

Figure S14: 1H NMR spectra of compound **15**

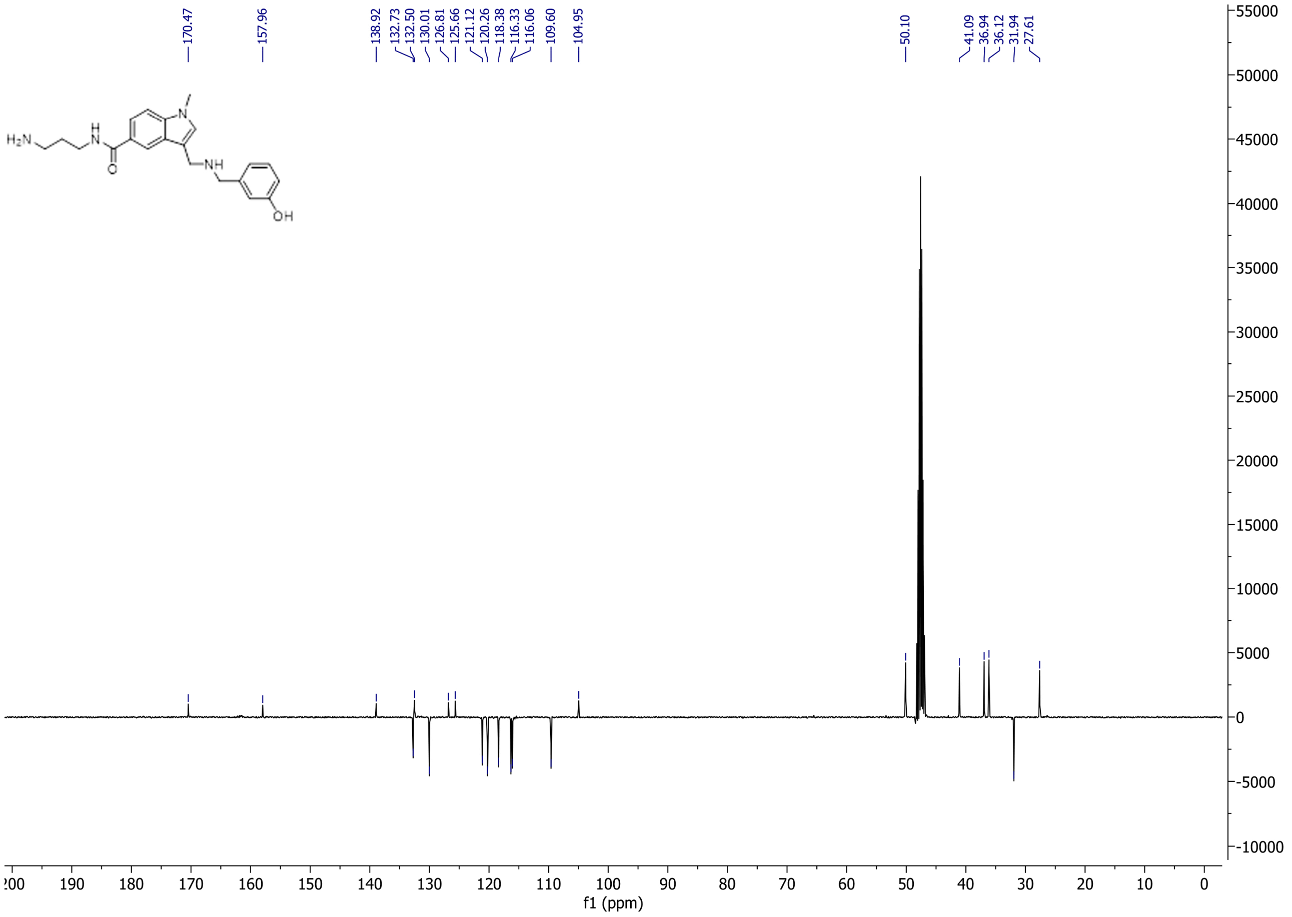

Figure S15: DEPT spectra of compound **15**

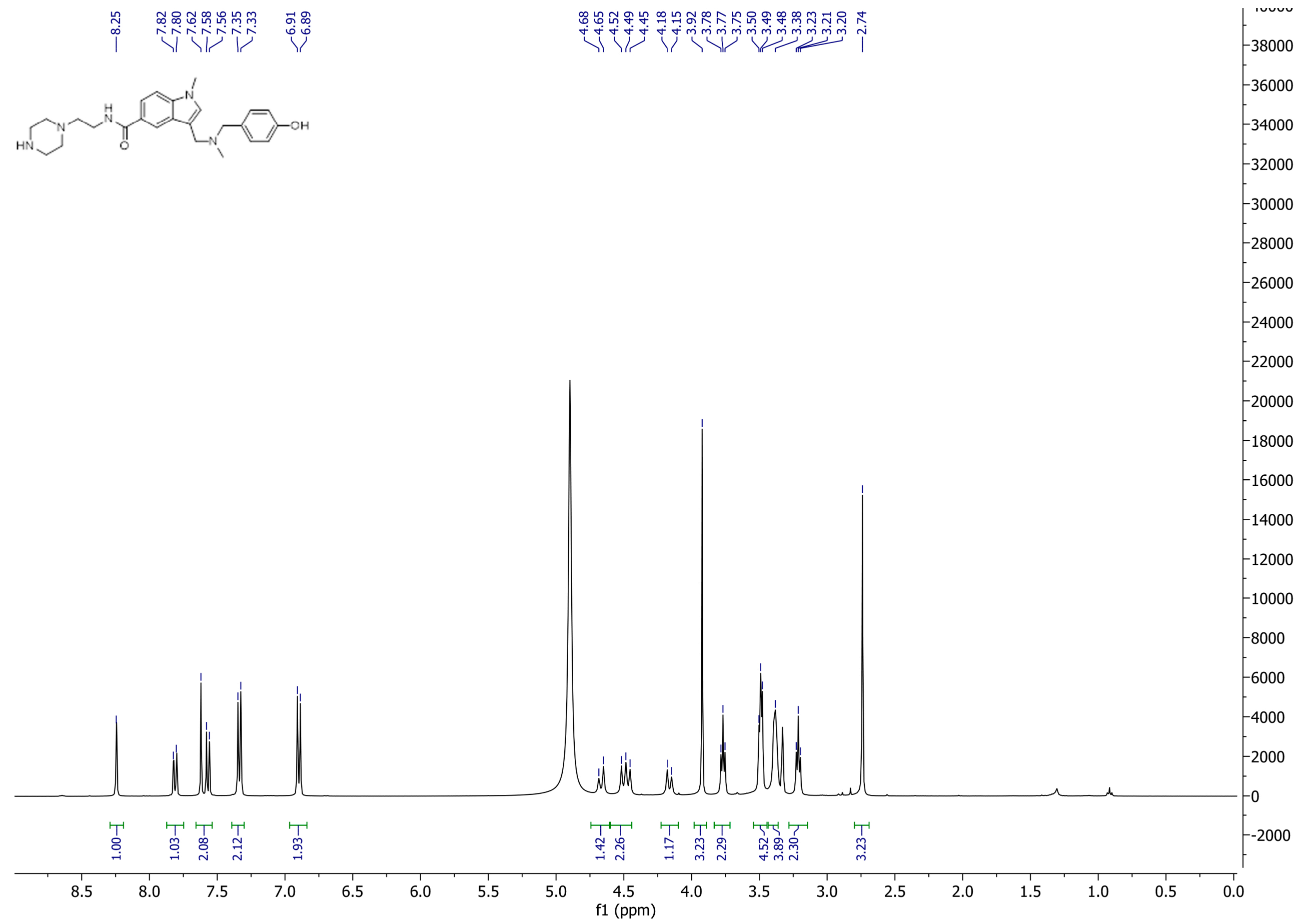

Figure S16: 1H NMR spectra of compound **20**

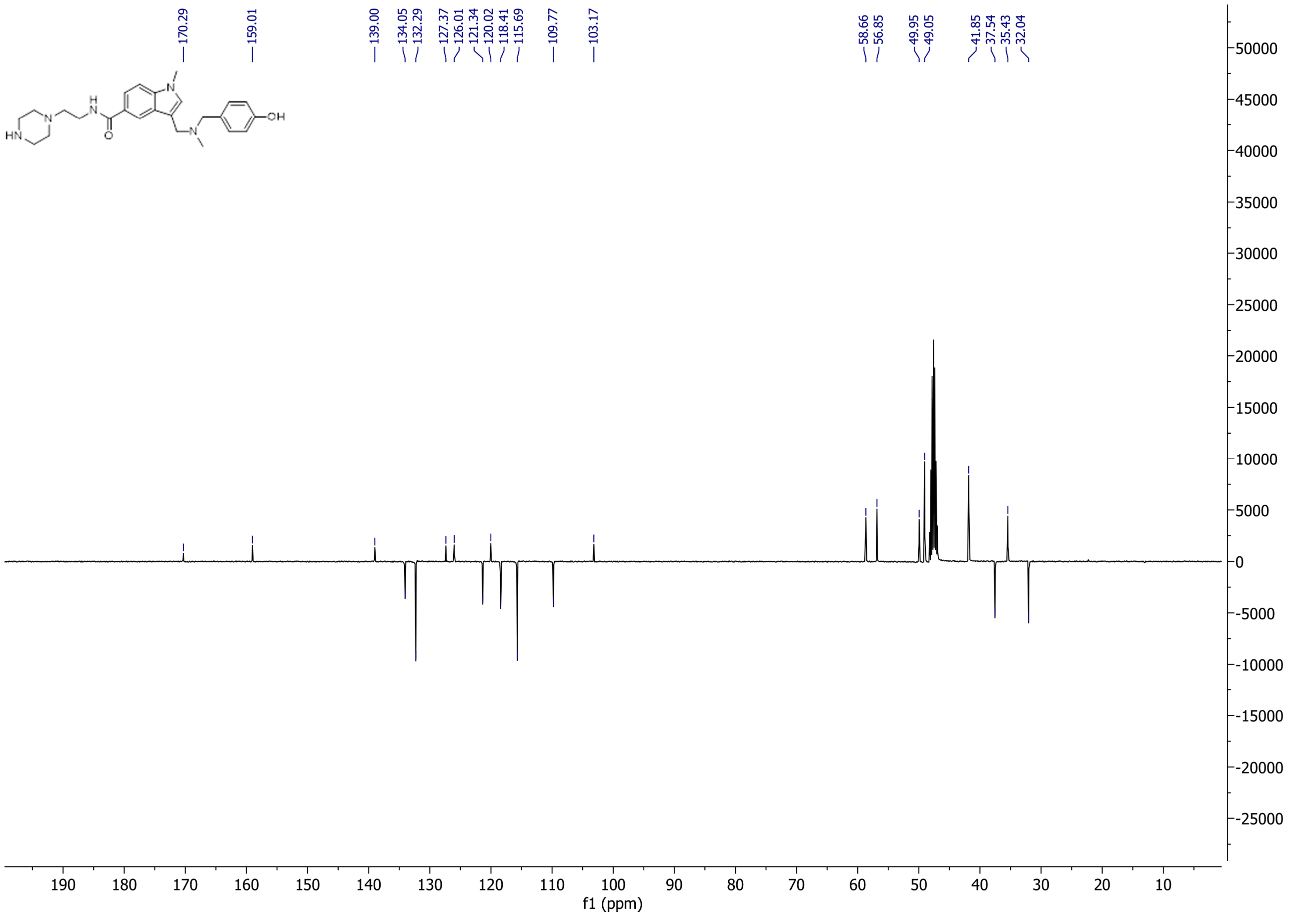

Figure S17: DEPT spectra of compound **20**

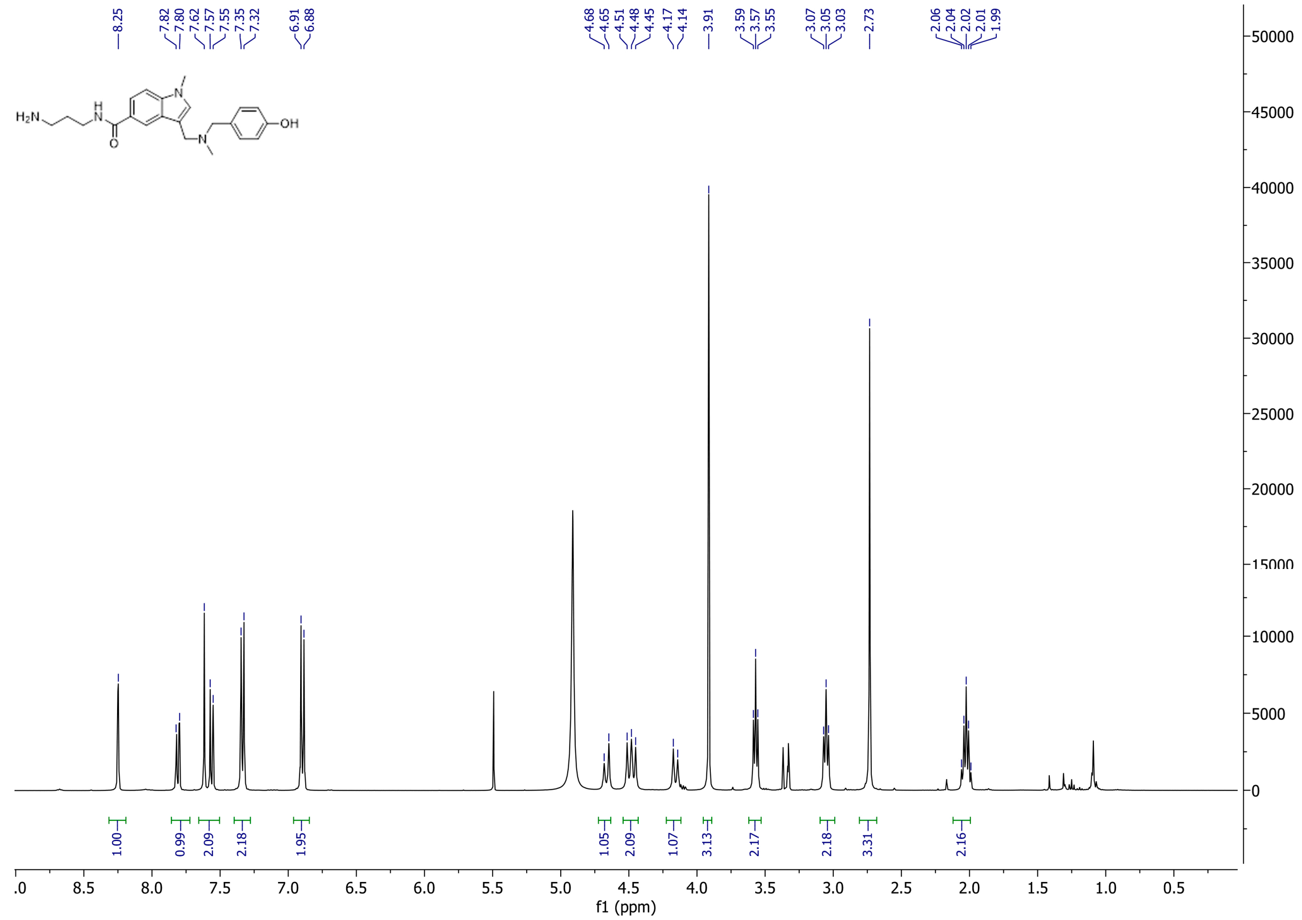

Figure S18: 1H NMR spectra of compound **21**

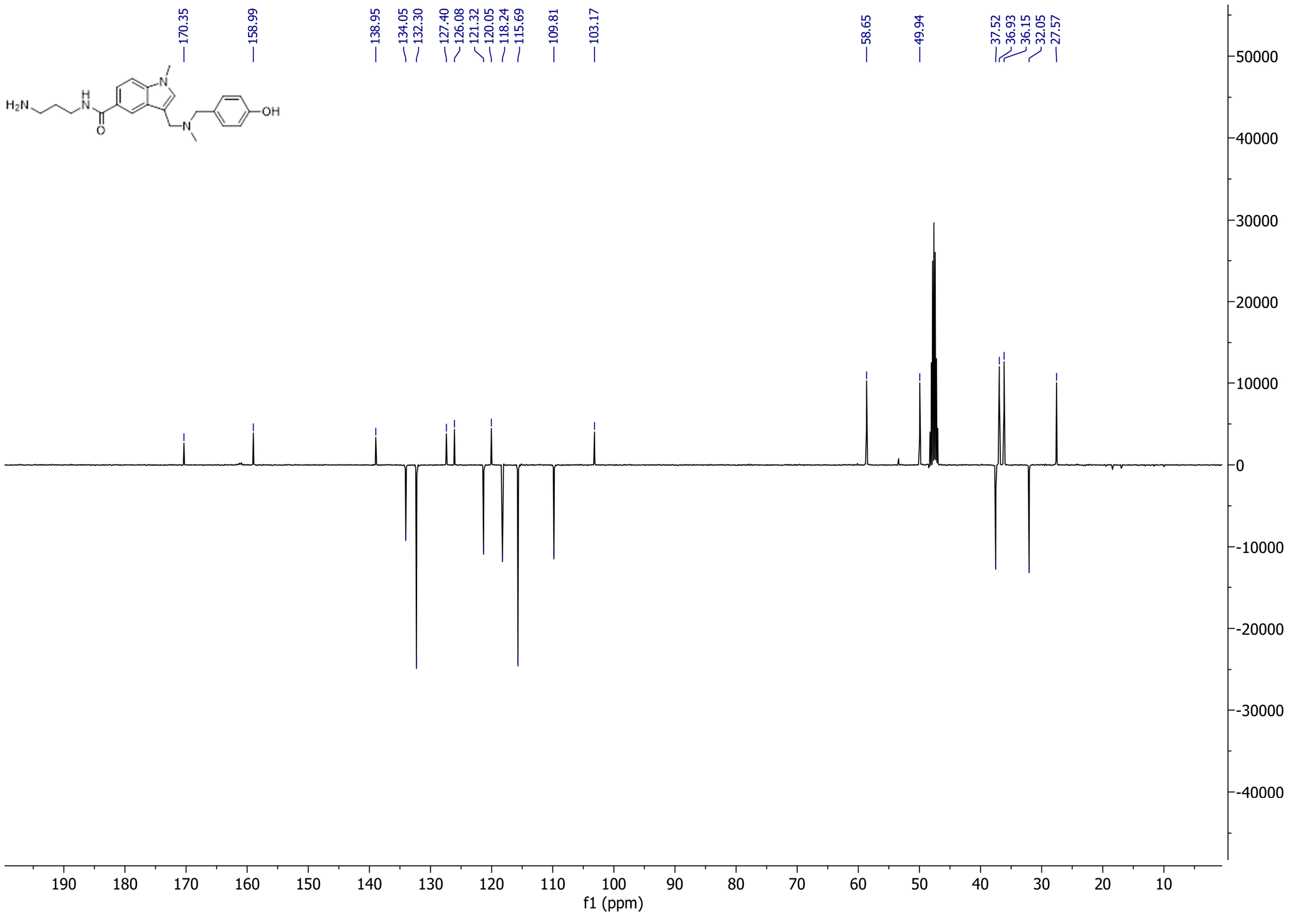

Figure S19: DEPT spectra of compound **21**

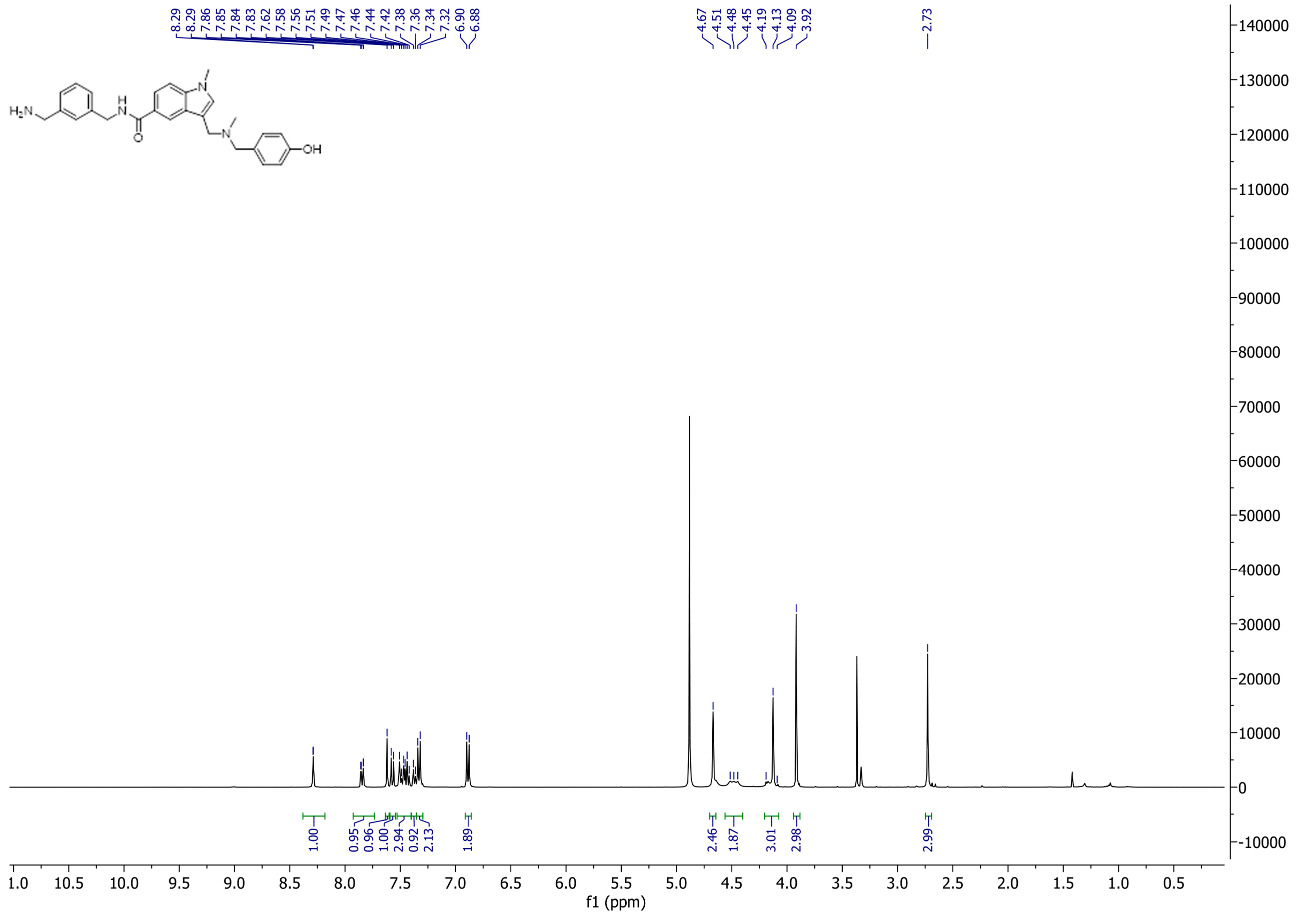

Figure S20: 1H NMR spectra of compound **22**

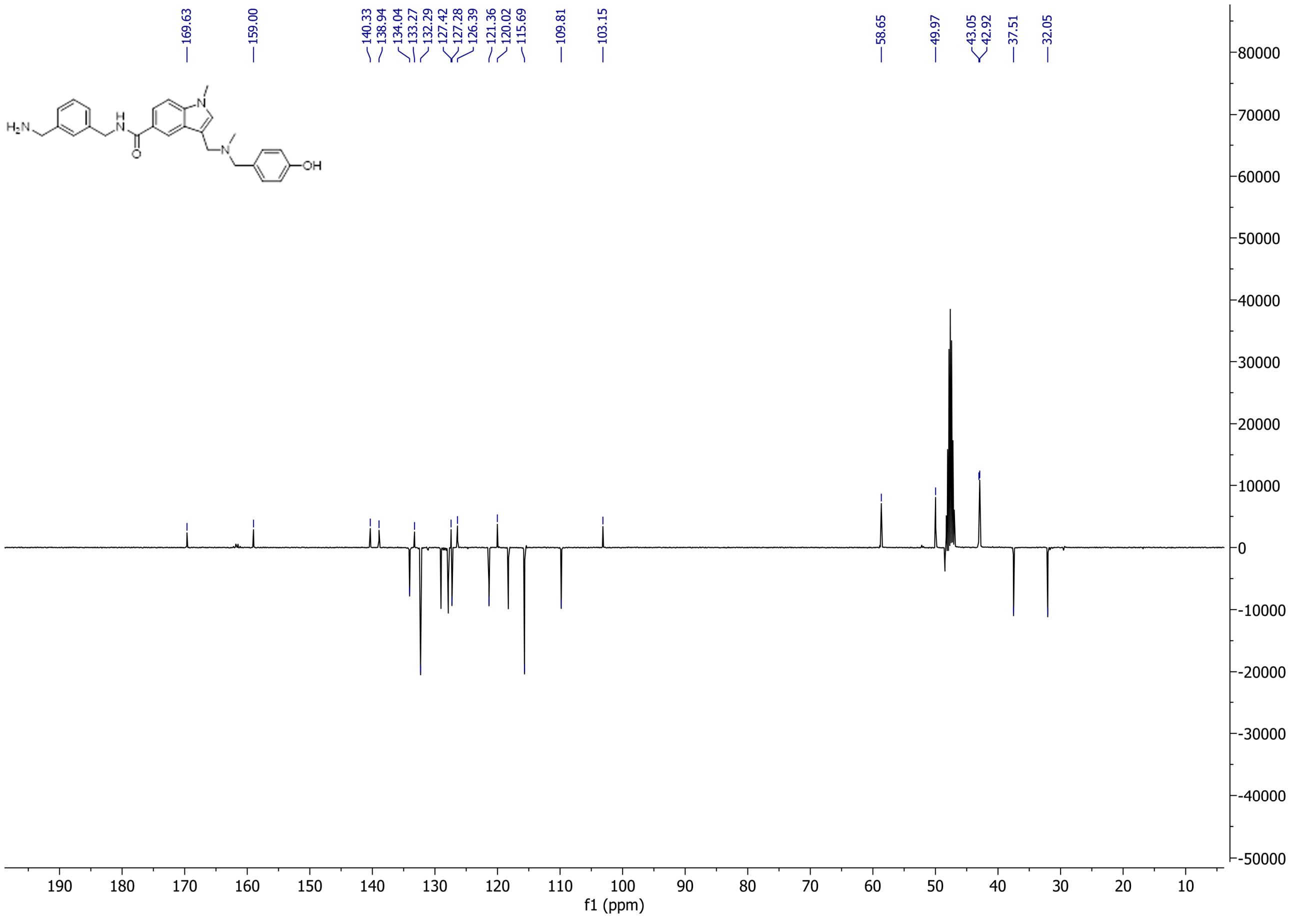

Figure S21: DEPT spectra of compound **22**

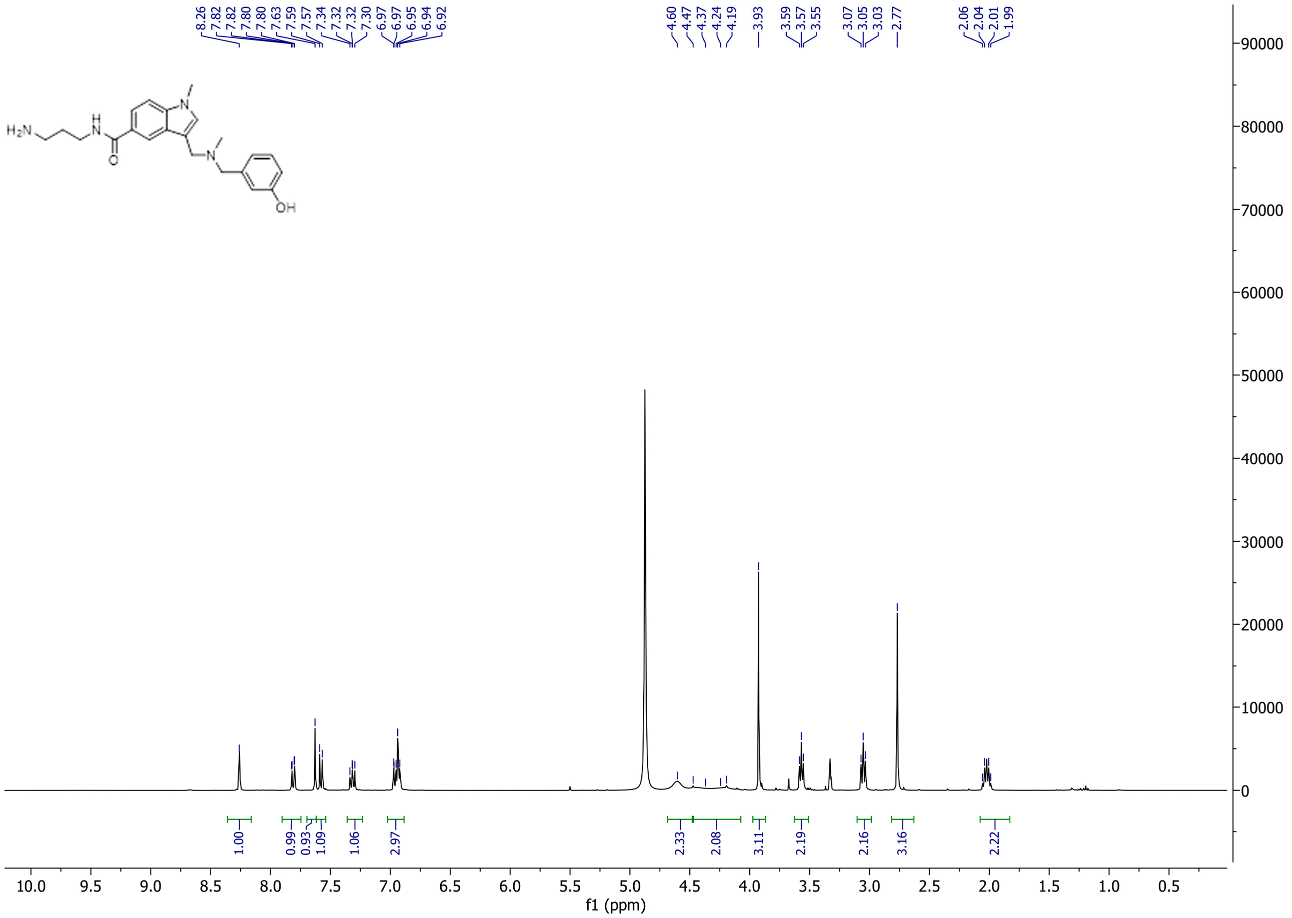

Figure S22: 1H NMR spectra of compound **23**

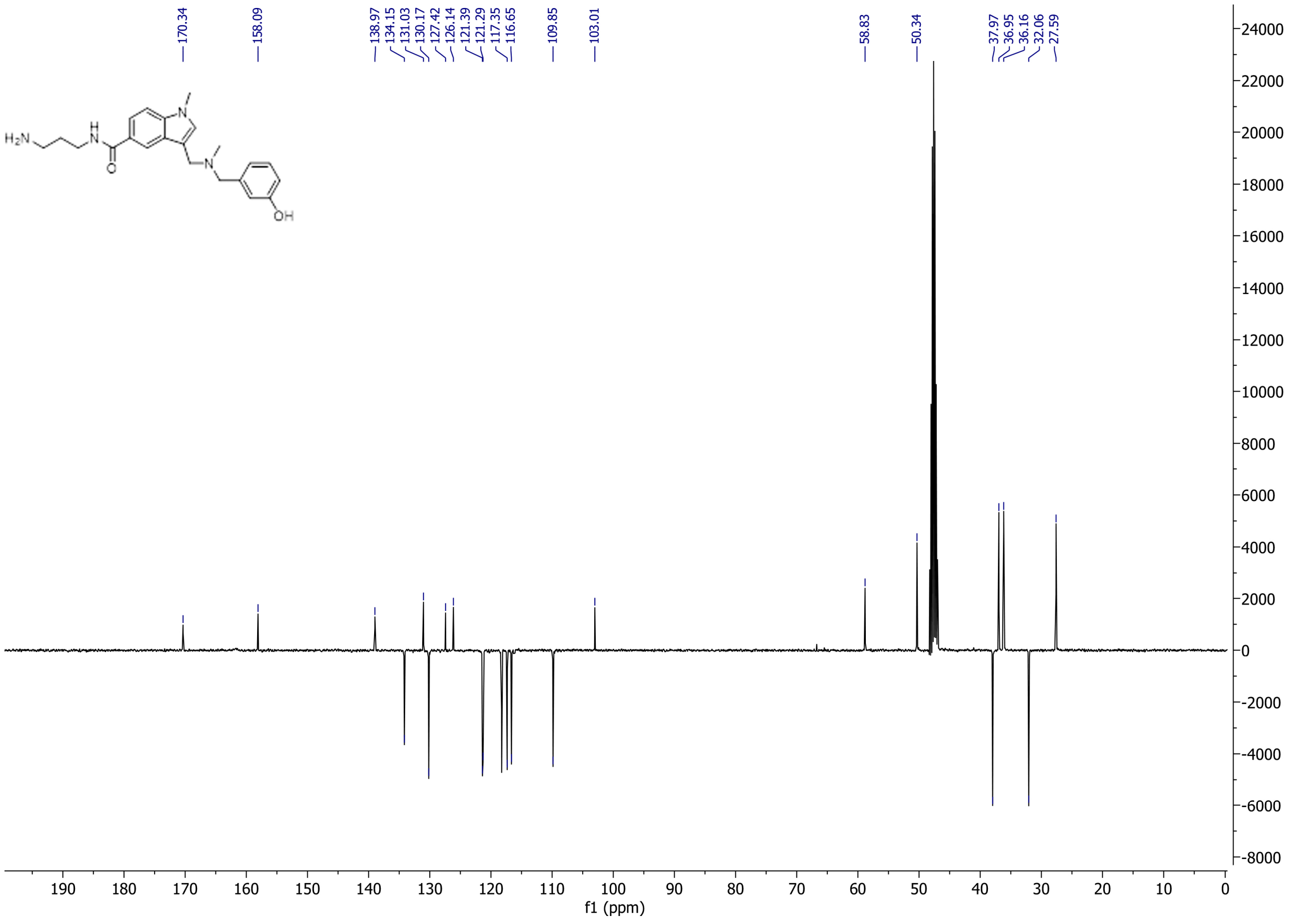

Figure S23: DEPT spectra of compound **23**

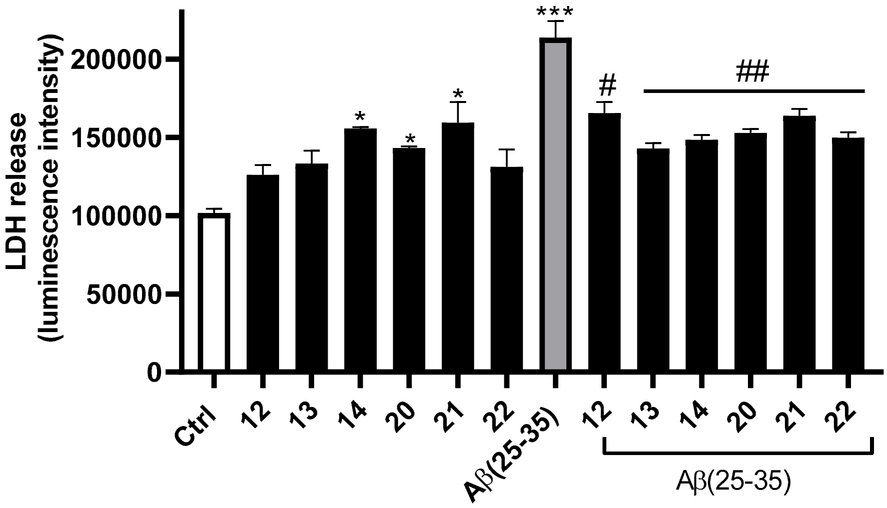

Figure S24. LDH assay performed on SH-SY5Y cells treated with 30 µM indole-based compounds and Aβ(25-35) 40 µM. Results are showed as mean ± standard deviation (SD) from three independent experiments. *, *** denote respectively $p < 0.05$ and $p < 0.001$ vs. Ctrl; #, ## denote respectively $p < 0.05$ and $p < 0.001$ vs. Aβ(25-35).

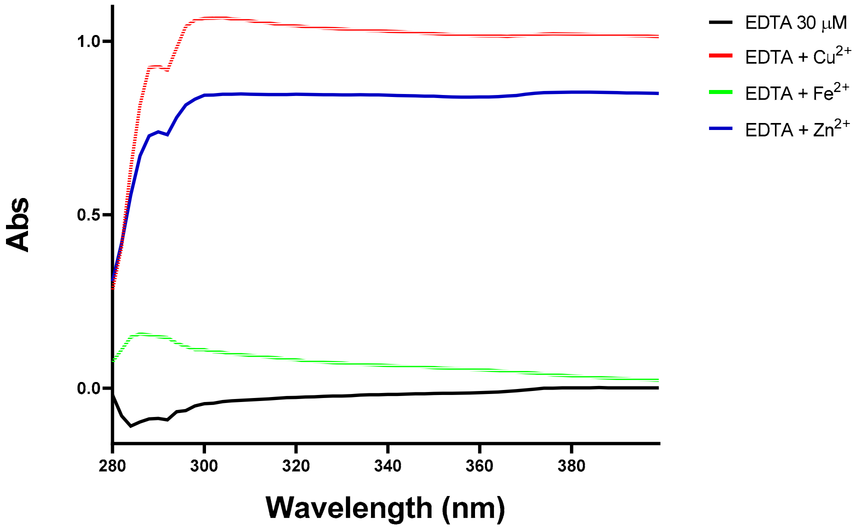

Figure S25. UV spectra (in range 280 to 400 nm) of positive ctrl EDTA (30 μM) alone and in the presence of 40 μM $FeSO_4$, $FeCl_3$ and $CuSO_4$.
